# Perception of incongruent audiovisual English consonants

**DOI:** 10.1371/journal.pone.0213588

**Published:** 2019-03-21

**Authors:** Kaylah Lalonde, Lynne A. Werner

**Affiliations:** Department of Speech and Hearing Sciences, University of Washington, Seattle, Washington, United States of America; University of Iowa, UNITED STATES

## Abstract

Causal inference—the process of deciding whether two incoming signals come from the same source—is an important step in audiovisual (AV) speech perception. This research explored causal inference and perception of incongruent AV English consonants. Nine adults were presented auditory, visual, congruent AV, and incongruent AV consonant-vowel syllables. Incongruent AV stimuli included auditory and visual syllables with matched vowels, but mismatched consonants. Open-set responses were collected. For most incongruent syllables, participants were aware of the mismatch between auditory and visual signals (59.04%) or reported the auditory syllable (33.73%). Otherwise, participants reported the visual syllable (1.13%) or some other syllable (6.11%). Statistical analyses were used to assess whether visual distinctiveness and place, voice, and manner features predicted responses. Mismatch responses occurred more when the auditory and visual consonants were visually distinct, when place and manner differed across auditory and visual consonants, and for consonants with high visual accuracy. Auditory responses occurred more when the auditory and visual consonants were visually similar, when place and manner were the same across auditory and visual stimuli, and with consonants produced further back in the mouth. Visual responses occurred more when voicing and manner were the same across auditory and visual stimuli, and for front and middle consonants. Other responses were variable, but typically matched the visual place, auditory voice, and auditory manner of the input. Overall, results indicate that causal inference and incongruent AV consonant perception depend on salience and reliability of auditory and visual inputs and degree of redundancy between auditory and visual inputs. A parameter-free computational model of incongruent AV speech perception based on unimodal confusions, with a causal inference rule, was applied. Data from the current study present an opportunity to test and improve the generalizability of current AV speech integration models.

## Introduction

Speech perception is inherently multimodal. In face-to-face communication, we automatically combine speech information from the face and voice. This automaticity is dramatically demonstrated in the popular McGurk illusion [1]. When presented an auditory /bɑbɑ/ paired with visual /gɑgɑ/, participants often perceive a fused /dɑdɑ/ that was not present in either modality. The McGurk illusion demonstrates that the brain integrates signals from across modalities into a single perceptual representation.

Of course, adults don’t always integrate incongruent speech information. Often, we are able to detect that stimuli do not match. For example, at certain temporal asynchronies, participants are able to tell that auditory and visual components of speech were not presented simultaneously (e.g., [2, 3]). These asynchronous speech signals result in decreased AV benefit [4–6] and decreased likelihood of fusing McGurk stimuli [2, 3], suggesting that AV speech perception varies depending on whether listeners attribute auditory and visual signals to the same source. The process of deciding whether two signals come from the same source is called causal inference [7]. It prevents perceivers from mistakenly integrating information from different talkers. Thus, causal inference is an essential part of AV speech perception [8].

Causal inference in AV speech has been studied predominately in the perception of asynchronous speech [2,3]. Research on the perception of *synchronous* AV speech typically assumes that the auditory and visual information will be integrated and focuses on how the information is combined into a single representation or category [8]. Typically, participants are asked to report what they *heard* and/or respond using a closed-set format. Therefore, when a participant’s response to an incongruent stimulus matches the auditory component, it’s not possible to determine whether 1) they were aware of the mismatch and reported what they heard or 2) they perceived a single AV stimulus consistent with the auditory component. In fact, when asked at the end of one such experiment, at least half of the participants stated that they detected mismatched stimuli [9]. This suggests that we might see a different pattern of perceptual responses if we ask participants what they *perceived*—rather than what they *heard*—and give them the option to report that they perceived the auditory and visual signals as coming from two different sources.

Magnotti and Beauchamp [8] recently demonstrated the utility of incorporating causal inference into a computational model of incongruent AV speech perception. They designed a causal inference model of multisensory speech (CIMS) and compared it to an otherwise identical non-CIMS model without a causal inference component. Both models were used to predict what participants heard when presented various combinations of auditory and visual voiced stops (/bɑ/, /dɑ/, and /gɑ/). The non-CIMS model assumed that the auditory and visual stimuli came from a single source. The percept was modeled as the integrated AV representation. The CIMS model assumed that participants would report hearing the auditory component when they perceived the auditory and visual signals as coming from different sources. The CIMS percept was modeled as the weighted average of the integrated AV representation and the auditory representation, with the weighting determined by the likelihood that the auditory and visual signals came from the same vs. different sources. The likelihood estimates were based on the content and natural statistics of the AV speech. The CIMS and non-CIMS models performed equally well when predicting behavioral responses to congruent AV speech, but the CIMS model was much better than the non-CIMS model at predicting human perception of incongruent AV speech. Thus, incorporating causal inference improved the model’s ability to predict behavioral responses to incongruent AV speech.

The studies reviewed above demonstrate that causal inference is an important step in AV speech perception. One goal of the current study is to improve our understanding of causal inference in perception of synchronous—but incongruent—auditory and visual speech. Specifically, the current study identifies which combinations of incongruent English consonants participants judge to have a common source and which combinations result in awareness that the auditory and visual signals do not match.

Causal inference has not yet been directly explored in synchronous AV speech. However, traditional research on AV speech integration provides a basis for forming hypotheses about what aspects of auditory-visual speech will predict participants’ causal inference judgments. Specifically, previous data show that the perception of a particular pairing of incongruent auditory and visual consonant depends on 1) which articulatory features differ across modalities and 2) the relative reliability of the auditory and visual signals for conveying each articulatory feature [10–12]. For example, in quiet listening conditions, stop consonants differing only in place of articulation (e.g., /bɑ/ and /gɑ/) result in fused (e.g., /dɑ/) and combined (e.g., /bga/) percepts [1], because place information is reliable in both auditory and visual domains. However, when auditory place of articulation cannot be reliably distinguished due to noise, the visual signal determines place of articulation [11]. Similarly, because the visual signal transmits very little voicing information [13], perception of incongruent pairs differing only in voicing is dominated by the auditory syllable.

Predicting responses is more complicated when the auditory and visual signals differ along multiple articulatory dimensions. In fact, only a few studies have attempted such predictions [9, 14]. MacDonald and McGurk [14] hypothesized that the manner and voice of incongruent AV speech are determined by the auditory consonant, and the place of incongruent AV speech is determined by the visual consonant. For example, an auditory front nasal /m/ paired with a visual back stop /k/ would result in a back (voiced) nasal /n/. To test this hypothesis, they collected perceptual responses to incongruent auditory-visual combinations of the syllables /pɑ/, /bɑ/, /tɑ/, /dɑ/, /kɑ/, /gɑ/, /mɑ/, and /nɑ/. The hypothesis held for auditory-front/visual-front, auditory-back/visual-back and auditory-front/visual-back consonant pairs, but was less useful for explaining auditory-back/visual-front consonant pairs. Nevertheless, the manner-place hypothesis provides a framework from which to assess causal inference.

We might expect participants to perceive auditory-back/visual-front consonant pairs as resulting from two separate sources. A closer look at MacDonald and McGurk’s [14] results supports this hypothesis. Participants’ responses were more likely to be consistent with the auditory consonant when visual front stop consonants (/pɑ/, /bɑ/) were paired with auditory back stop consonants (/kɑ/, /gɑ/) than when paired with auditory middle stop consonants (/tɑ/, /dɑ/). In other words, with more extreme differences between the place of articulation of the auditory and visual stops, participants were more likely to report the auditory component. Given that participants were asked to report what they *heard*, it is possible that they were sometimes aware of the mismatch and reported the auditory component.

Jiang and Bernstein [9] hypothesized that the patterns of perceptual responses to incongruent AV speech depend on the learned correspondence between congruent auditory and visual speech. To investigate what perceivers have learned about the relationship between auditory and visual speech signals, these researchers characterized the physical relationship between two auditory speech signals (/bɑ/ and /lɑ/) and seven visual speech signals, then related these physical characteristics to perceptual responses. As in other studies, participants were asked to report what they heard. Fusion responses were more common when there was a low correspondence between the physical characteristics of the auditory and visual signals; auditory responses were more common when there was a high correspondence between the physical characteristics of the auditory and visual signals. These results suggest that the degree of redundancy between incongruent auditory and visual speech signals affects patterns of perceptual responses to incongruent AV speech. Visual speech influenced perception of auditory /bɑ/ more than auditory /lɑ/, suggesting an important role of auditory place and/or manner in determining patterns of perceptual responses to incongruent AV speech. Responses were highly stimulus-specific, suggesting that more patterns will emerge from this study’s examination of responses to the full set of incongruent AV English consonants.

As noted above, at the end of Jiang and Bernstein’s [9] experiment, at least half of participants reported detecting mismatched stimuli, suggesting that we can gain a better understanding of the patterns of perceptual responses to incongruent AV speech by examining 1) causal inference and 2) the pattern of perceptual responses that occur after controlling for causal inference.

Based on the above research, we might expect participants’ causal inference judgments to depend on whether they differ on features that are reliably conveyed in both modalities (i.e., some differences in place and/or manner) and whether the visual information is salient (i.e., front place of articulation). Intuitively, we might also expect participants to be aware that the auditory and visual signals come from different sources when there is low redundancy between the auditory and visual signals (i.e., when the auditory and visual consonants are discriminable from one another in both the auditory and visual modalities).

To explore these hypotheses, we collected causal inference judgments using congruent and incongruent AV syllables and related these judgments to the reliability and salience of information in each modality and redundancy of information across modalities. More specifically, we relate causal inference judgments to 1) unimodal accuracy, 2) auditory and visual place of articulation, 3) the match/mismatch of auditory and visual articulatory features, and 4) patterns of unimodal consonant confusions. Additionally, for those consonant combinations that were judged as coming from a single source, we relate the same features to 1) responses consistent with the auditory signal, 2) responses consistent with the visual signal, and 3) responses that were not consistent with either the auditory or visual input (e.g., fused and combined responses).

We expect that data from the current study will be valuable as researchers begin to incorporate causal inference into computational models of AV speech perception. To demonstrate the utility of our data, we applied one quantitative model to incongruent AV speech, the Fuzzy Logic Model of Perception (FLMP) [15]. The FLMP predicts response to AV speech based on confusions among unimodal auditory and unimodal visual consonants. We modified the FLMP to incorporate causal inference judgments and hypothesized that—with this adjustment—the FLMP would predict incongruent AV speech perception as well as it predicts congruent AV speech perception.

## Materials and methods

### Participants

Nine adult subjects (8 female) between 18 and 30 years of age participated in the experiment. However, one subject’s incongruent AV data were excluded because they forgot or ignored the instructions to report what they perceived, rather than what they heard. Unlike other participants who made a high proportion of auditory responses, this participant’s proportion of auditory responses increased drastically between the first day of testing and later test sessions. All subjects reported normal hearing, normal or corrected-to-normal vision, and native English backgrounds. All subjects passed a hearing screening.

### Stimuli

The stimuli were professional AV recordings of a 46-year-old female white, non-Hispanic, native English speaker with a master’s degree in Theatre. The speaker was instructed to look into the camera before beginning to speak each syllable, read the syllables (presented both orthographically and in IPA) from a teleprompter, and try to keep a constant duration, amplitude, pitch, and cadence across syllables. The syllables were presented on the teleprompter in a random order, so that any fatigue effects would be distributed across syllables. Five tokens of each consonant-vowel (CV) syllable were recorded. The 69 CVs were the consonants /p, b, m, w, r, f, v, θ, ð, ʃ, ʒ, tʃ, dʒ, s, z, t, d, n, j, k, g, h, l/ with the vowels /ɑ, i, u/. Two high quality productions of each CV were chosen as test stimuli. Stimuli were edited in Final Cut Pro (Version 10.0.6) and Adobe Audition (Version 6). Each movie file began 500 ms before the onset of the consonant sound and ended at the end of the vowel. Because all video files began 500 ms before the onset of the speech sound, the audio and video portions of incongruent AV stimuli were matched to the onset of the sound. In other words, the temporal relationship between the onset of the mouth movement and onset of the sound was preserved for the visual stimulus in the AV incongruent condition. The level of each syllable was adjusted to equate the total RMS power across all of the vowels, while ensuring that no peak-clipping resulted from amplifying the signals. The final set of stimuli are available for download in the following location: https://osf.io/6gk7p

The auditory syllables were presented to the right ear in quiet via an ER-2 insert earphone. The level of the vowels was 70 dB SPL (flat weighting), calibrated using a Zwislocki 2-cc coupler. The visual stimulus consisted of the full face, down to the shoulders; the face extended 8.25 x 6 inches on a 27-inch monitor. Participants sat approximately 2.5 feet from the monitor.

### Procedures

All testing took place in a double-walled sound booth. Participants watched and/or listened to the syllable presented and indicated what they perceived. During the first portion of testing, participants were presented a mixture of congruent and incongruent AV syllables. They were then presented auditory-only, visual-only, and congruent AV syllables, with modality order counterbalanced across participants. On each trial, participants saw a video and/or heard the audio of a CV syllable. Then, a text entry box and a drop-down list of the response options appeared on the screen. Participants responded by typing what they perceived or choosing their response from the drop-down list. The drop-down list included the option to indicate that the auditory and visual syllables did not match and the option to type something else that was not on the list. After a few trials with the drop-down list, all participants chose to type their responses. As a short-hand, participants could type “QQ” in the text-entry box to indicate that the auditory and visual syllables did not match. Testing was self-paced; participants clicked “Next” to advance to the next stimulus.

Before testing, participants received a list of potential responses, with orthographic representations of each consonant paired with the vowel being tested (e.g., “boo,” “sah,” and “ree”) and words that began with each syllable. There were two exceptions: 1.) The consonant /ʒ/ does not occur in a word-initial position in English, so we used the example, “end of ‘rouge,’ middle of ‘measure.’” 2.) To differentiate between /ð/ and /θ/, we compared “the ‘th’ in ‘the’ and ‘that’” to “the ‘th’ in ‘thing.’” Some consonants have different phonetic and orthographic representations. Therefore, we used the following: “ch” for /tʃ/, “j” for /dʒ/, “sh” for /ʃ/, “th” for /ð/, “thh” for /θ/, “zh” for /ʒ/, and “y” for /j/. We consider the response open-set, because the list included all syllable-initial English consonants and because participants could also choose a response that was not on the list, such as a syllable with a consonant cluster (e.g., “mbah” or “sfoo”), or indicate that the auditory and the visual syllables did not match. If the participants perceived a combination of two stimuli (i.e., AV “mbah”), they were instructed to type that response. If instead, they perceived the two components as independent (i.e., auditory “mah” and visual “bah), they were instructed to report a mismatch. Participants were instructed to use the same orthography to report any percept that was not included in the list (i.e., a consonant cluster). Before beginning testing, the experimenter read the list of response options to the participant and checked that they understood.

The AV-incongruent condition included a total of 7,935 trials per participant (529 consonant pairs x 3 vowels x 5 repetitions). Stimuli were presented in 529-trial test blocks, with vowel held constant. Each of the 23 auditory consonants was paired with each of the 23 visual consonants once in each block. This resulted in 23 congruent and 506 incongruent AV syllables. Participants completed five blocks with the same vowel context before continuing to the next vowel. Vowel order was counterbalanced across participants, and AV consonant pair order varied randomly within each block. Participants were informed that the auditory and visual stimuli would match on some trials, but not on others. They were instructed to attend to both the auditory and visual stimuli and to report what they perceived.

Auditory-only, visual-only, and congruent AV conditions each included 414 trials (23 consonants x 3 vowels x 6 repetitions). Stimuli were presented in 138-trial test blocks. In each block, modality and vowel were held constant, and the 6 repetitions of the 23 consonants were presented in a random order. Participants completed testing in all 3 modalities with the same vowel before moving to the next vowel. Modality and vowel order were counterbalanced across participants. During auditory-only testing, a neutral image of the talker with her mouth closed was presented throughout each stimulus interval. During visual-only testing, the videos were presented with no sound. In the congruent AV condition, auditory and visual speech were synchronous and congruent. We verified the temporal alignment of AV stimuli presented through our experimental system by examining the alignment of simultaneously presented auditory clicks and visual flashes using an oscilloscope and a photosensor.

Participants completed 12.6 to 21.8 hours (mean = 16 hours) of testing in 60- to 90-minute sessions. They received $15 per hour of testing at the end of the experiment. The experiment used dedicated software in Python (version 2.6.6). Data were processed and analyzed using MATLAB (Mathworks, Natick, MA, USA).

## Analysis and results

In all of the analyses and figures below, data represent the mean across the three vowel contexts. We would expect to observe some differences in consonant perception as a function of vowel context, as—for example—many visual consonants are easier to discriminate with the open vowel /ɑ/ than the close, back vowel /u/ [13] (See also, Table 1). This could prompt participants to rely more on the auditory stimulus in the /u/ context than in the /ɑ/ context. Therefore, analyses of each individual vowel context are included in the S1 Appendix. In general, visual-only accuracy and the proportion of each response type varied across vowel contexts. However, the relationship between predictive variables and proportions of mismatch, auditory, and Other responses was typically the same across vowels. Some vowel-related differences were found for visual responses, likely due to their relatively small representation of visual responses in the data.

**Table 1:** Viseme categories created using hierarchical cluster analysis, with minimum 70% cluster criterion.

Category Description	vowel /ɑ/		vowel /i/		vowel /u/	
bilabial	/p, b, m/	98.2%	/p, b, m/	100%	/p, b, m/	95.1%
labiodental	/f, v/	97.2%	/f, v/	99.1%	/f, v/	93.5%
interdental	/θ, ð/	87%	/θ, ð/	97.2%	/θ, ð/	91.7%
/w/	/w/	100%	/w/	98.2%	/w/	88.9%
/r/	/r/	81.5%	/r/	90.7%	/r/	85.2%
/l/	/l/	94.4%	/l/	96.3%	/l,	94.4%
postalveolar fricatives/affricates	/ʃ, tʃ, ʒ, dʒ/	94%	/ʃ, tʃ, ʒ, dʒ/	90.7%	ʃ, tʃ, ʒ, dʒ,	
alveolar stops/fricatives	/s, z, t, d/	86.1%	/s, z, t, d,	75.3%	s, z, t, d,	
palatal, velar, pharyngeal, and /n/	/k, g, h, j, n/	86.3%	k, g, h, j, n/		k, g, h, j, n/	

*Note*. The same categories are derived using a minimum 75% cluster criterion.

### Perception of auditory-only and congruent AV speech

**Fig 1:**
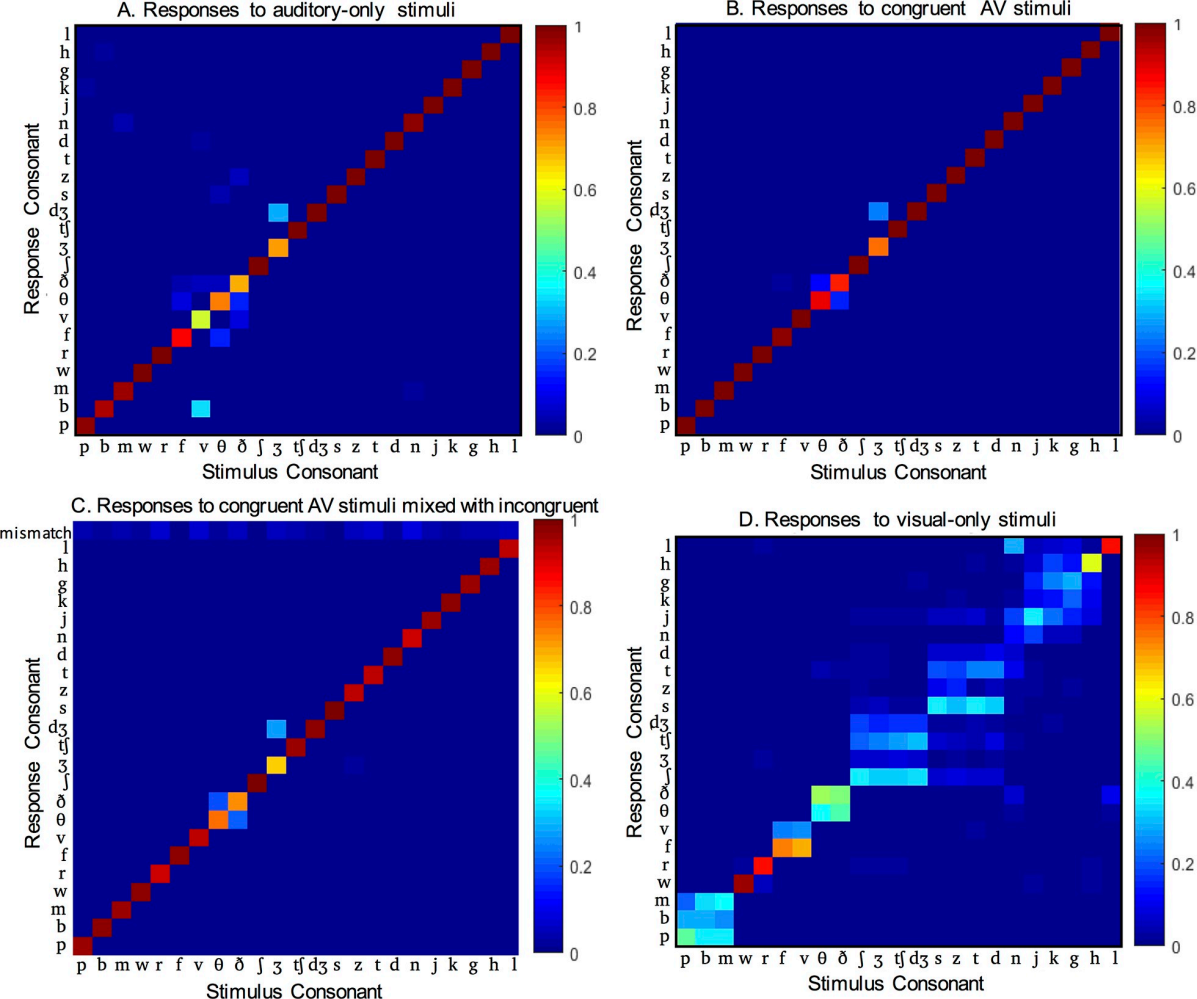
Responses to unimodal and congruent AV consonants. (A) Responses to auditory-only consonants. (B) Responses to congruent AV consonants. (C) Responses to the same congruent AV consonants when randomly presented amidst the incongruent stimuli. (D) Responses to visual-only consonants. Responses are averaged across participants and vowel contexts.

Fig 1 shows the results for the auditory-only, visual-only, and AV-congruent conditions, combined across subjects and vowel contexts. As we would expect for clear speech in quiet, participants made few errors in identifying auditory-only consonants (M = 93.2%, S.D. = 2.95) and congruent AV consonants (M = 97.4%, S.D. = 2.92%). Even with near ceiling performance, however, there was a significant AV benefit, *t* = 6.0084; *p* = 0.0003; df = 8.

The few errors participants made in auditory-only and congruent AV conditions were not surprising. Consistent with previous literature [16], the most common auditory errors were confusions among fricatives and substituting /b/ for /v/ (Fig 1A). A subset of participants also substituted /dʒ/ for /ʒ/. The addition of visual information eliminated the /b/-/v/ confusions and decreased confusions among some fricatives (Fig 1B). However, confusions between the fricatives /ð/ and /θ/ remained common, and the same subset of participants continued to substitute /dʒ/ for /ʒ/ in AV congruent conditions.

Congruent AV syllables were also presented mixed with the incongruent AV syllables (Fig 1C). Participants made 4.8% more errors on congruent trials randomly presented amidst the incongruent stimulus trials than when only congruent syllables were presented. The additional errors were reports that 3.91% of congruent syllables did not match when presented amidst with the incongruent stimulus trials (“mismatch” row, Fig 1C). The remaining errors were similar to those made when only congruent syllables were presented.

### Perception of visual-only speech

Not surprisingly, participants were much poorer at identifying consonants based on only visual speech (M = 38.3%, S.D. = 5.85%) (Fig 1D). We were interested in the pattern of visual consonant identification errors, because we hypothesized that these patterns would predict responses to incongruent AV speech. To characterize the visual-only error patterns, we created clusters of consonants that are visually confusable. These consonant clusters are called viseme categories [17]. In previous studies, viseme categories have been operationally defined as groups of consonants for which a minimum of 70% or 75% of responses to consonants in a viseme category are consonants in the viseme category [13, 18–20]. For example, if /p/, /b/, and /m/ form a viseme category, at least 70% of responses to a visual /p/ are /p/, /b/, and /m/.

To define the viseme categories present in our data, we used agglomerative hierarchical cluster analysis. Previous data shows that viseme clustering depends on vowel context [13]. Therefore, we completed separate analyses for each of the three vowel contexts. Each input observation in the analysis consisted of a vector representing the total response to all presentations of an individual CV syllable. In other words, if we averaged across vowel contexts, each column in Fig 1D would represent an input observation. We calculated the pairwise Euclidean distance between each pair of input observations to create a dissimilarity matrix, and then grouped the observations (consonants) into binary agglomerative hierarchical clusters. We progressively increased the number of clusters until the minimum proportion of within-cluster responses failed to reach the 70% criterion.

All clusters met the 70% criterion up to a maximum of 9, 8, and 6 clusters in the /ɑ/, /i/, and /u/ contexts, respectively. These clusters are shown in Table 1 and are generally consistent with previous studies [13]. Across all three vowel contexts, two of the approximants were identified with 81 to 100% accuracy (/w, r/) and formed distinct single-phoneme categories. In the /ɑ/ and /i/ contexts, /l/ also formed a distinct single-phoneme category. The other phonemes clustered largely as a function of place of articulation, with front places of consonant articulation and open/front vowels forming smaller, more distinct categories. There are bilabial, labiodental, interdental, palatal fricative/affricate, and alveolar-to-glottal clusters. Note that consonants in relevant figures are ordered so that members of the same viseme category are adjacent to one another.

### Perception of incongruent AV speech

Our primary goal was to examine which combinations of incongruent AV English consonants participants judge to have a common source and which combinations result in awareness that the auditory and visual signals do not match. Further, we aimed to examine patterns of perceptual responses to incongruent AV speech after controlling for causal inference. Based on traditional AV speech perception research, we hypothesized that causal inference judgments and responses to incongruent AV stimuli would depend on 1) the degree of redundancy between the auditory and visual consonants, 2) the reliability of information in each modality, 3) whether signal differ on features reliably conveyed in both modalities, and 4) the salience of visual information.

Consistent with these goals, we first examined those combinations of consonants for which participants were aware that the auditory and visual signals do not match (mismatch responses). Next, for those combinations that were judged as coming from a single source, we examine 1) responses consistent with the auditory signal, 2) responses consistent with the visual signal, and 3) responses that were not consistent with either the auditory and visual input (e.g., fused and combined responses).

**Fig 2:**
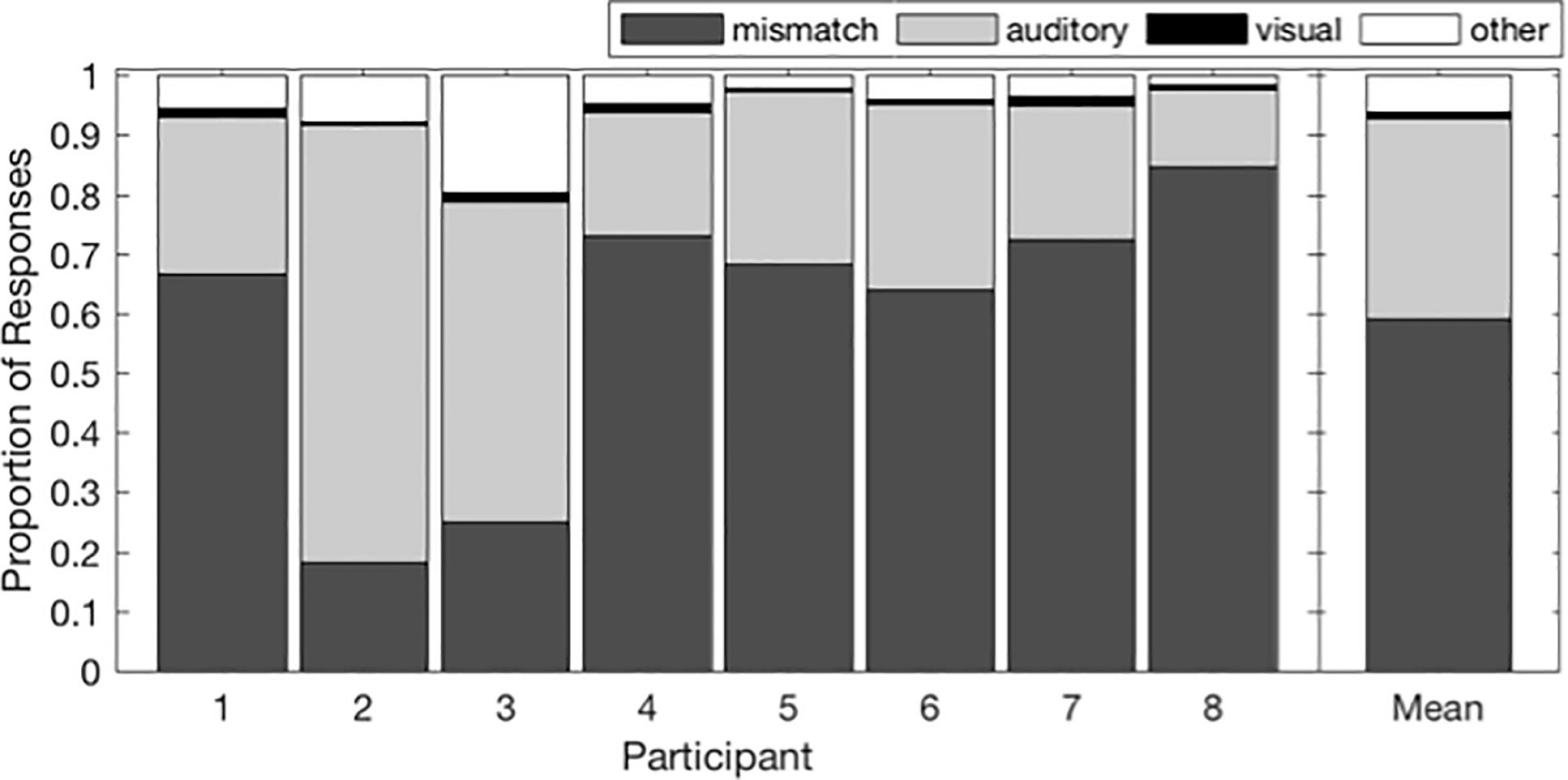
Proportions of each response type for incongruent syllables. Individual and mean proportion of mismatch, auditory, visual, and other responses to incongruent syllables.

The proportions of each type of response for each participant are shown in Fig 2; the group means are on the right. For the majority (59%) of incongruent AV consonant pairs, participants noticed the mismatch between auditory and visual signals. These consonant pairs violated participants’ expectations about what visual and auditory features should go together, and so they did not integrate the incongruent signals.

When participants integrated the auditory and visual information, they were most likely to report the auditory syllable (33.73%). They only reported the visual syllable 1.13% of the time. The remaining 6.11% of responses fell into the “other” category. “Other” responses include those that we traditionally think of as “integration” of incongruent AV speech signals: McGurk-like fused and combined percepts. This pattern of responses was consistent across 6 of 8 participants.

The other two participants had a smaller proportion of mismatch responses and a larger proportion of auditory responses. Note that despite the differences in overall response proportions, these subjects’ individual data follow nearly all of the group trends reported below with respect to the relationship between the predictive variables (viseme cluster, feature congruency, unimodal accuracy, place of articulation) and response types (mismatch responses, auditory responses, visual responses, and Other responses). The few exceptions were for visual and Other responses, for which responses were generally very few and highly variable, respectively.

### Analysis of responses to incongruent AV speech

**Fig 3:**
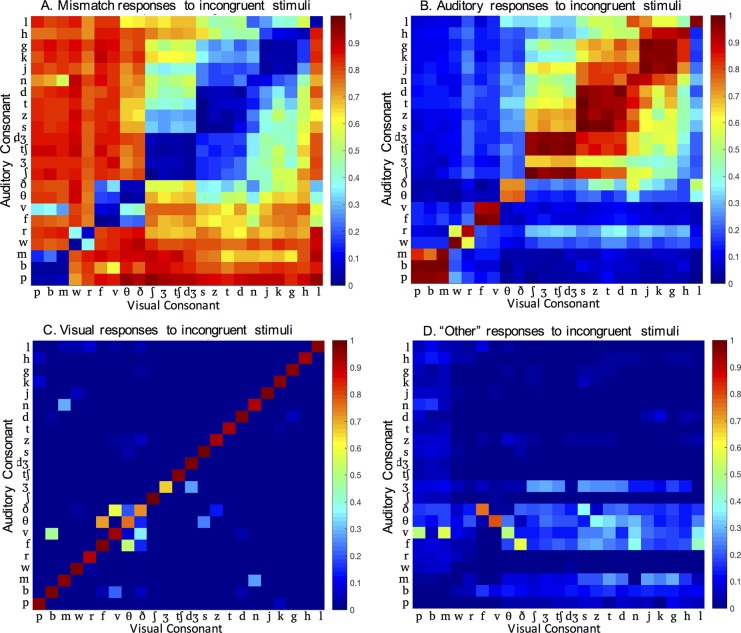
Responses to incongruent AV consonants. Proportion of (A) mismatch responses, (B) auditory responses, (C) visual responses, and (D) Other responses to incongruent AV syllables, as a function of auditory and visual consonant.

Fig 3 shows the proportion of auditory, visual, mismatch, and Other responses for each AV syllable pair. We conducted a series of analyses to determine what features predicted how participants perceive incongruent consonants. More specifically, we assessed what features were associated with mismatch, auditory, visual, and Other responses. We proceed by describing each of the analyses, then discussing the results for each response type.

#### Viseme clusters

**Fig 4:**
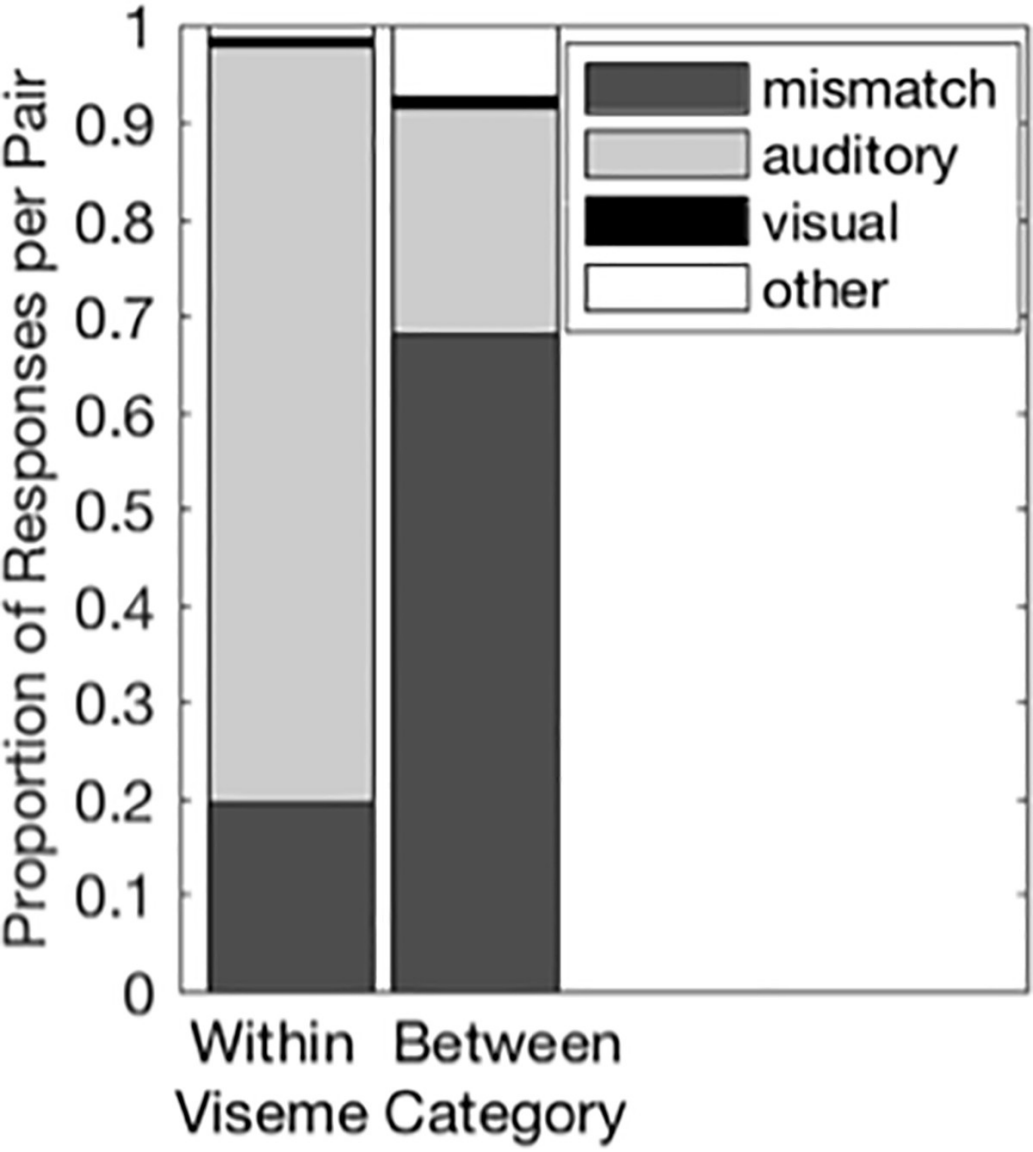
Responses to within- and between-cluster incongruent consonant pairs. Proportion of each response type to incongruent AV trials for within cluster and between cluster consonant pairs. Clusters are show in Table 1. See S1 Fig for means and standard deviations.

Intuitively, we expect participants to be aware that the auditory and visual signals come from different sources when there is low redundancy between the auditory and visual signals. Additionally, Jiang and Bernstein [9] demonstrated that the degree of redundancy between auditory and visual signals influences integrated responses (also see [21]). Therefore, we reasoned that responses would depend on whether the auditory and visual consonants were visually distinct. Consequently, we assessed whether responses differed for auditory and visual consonants in the same viseme cluster (Table 1) as compared to auditory and visual consonants in different viseme clusters. We used t-tests to compare the proportion of mismatch, auditory, visual, and Other responses for within- and between-viseme-cluster consonant pairs (Fig 4). Results are discussed for each response type below.

#### Unimodal accuracy

**Fig 5:**
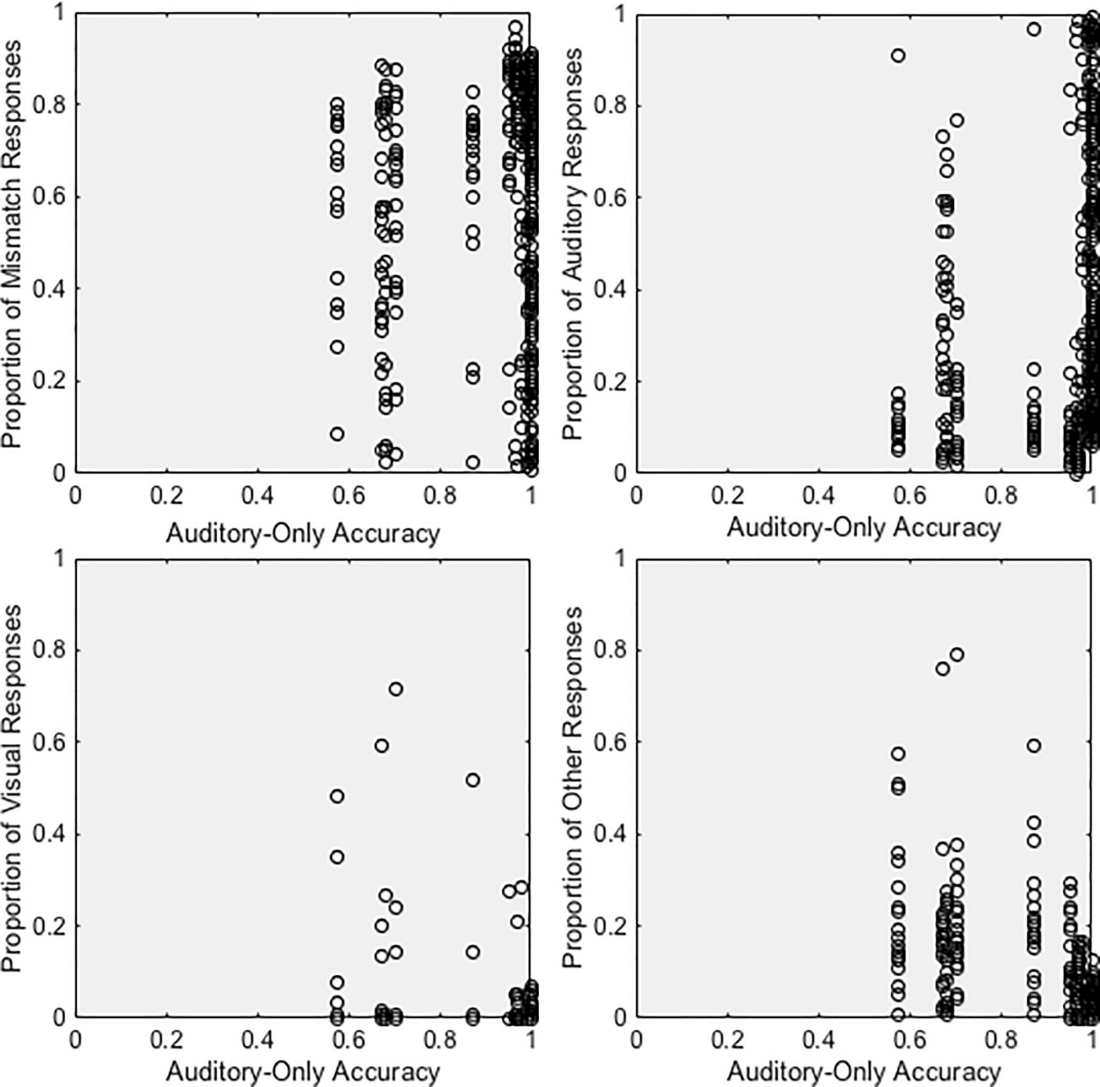
Responses as a function of auditory-only accuracy. Proportion of (A) mismatch responses, (B) auditory responses, (C) visual responses, and (D) Other responses as a function of auditory-only identification accuracy.

**Fig 6:**
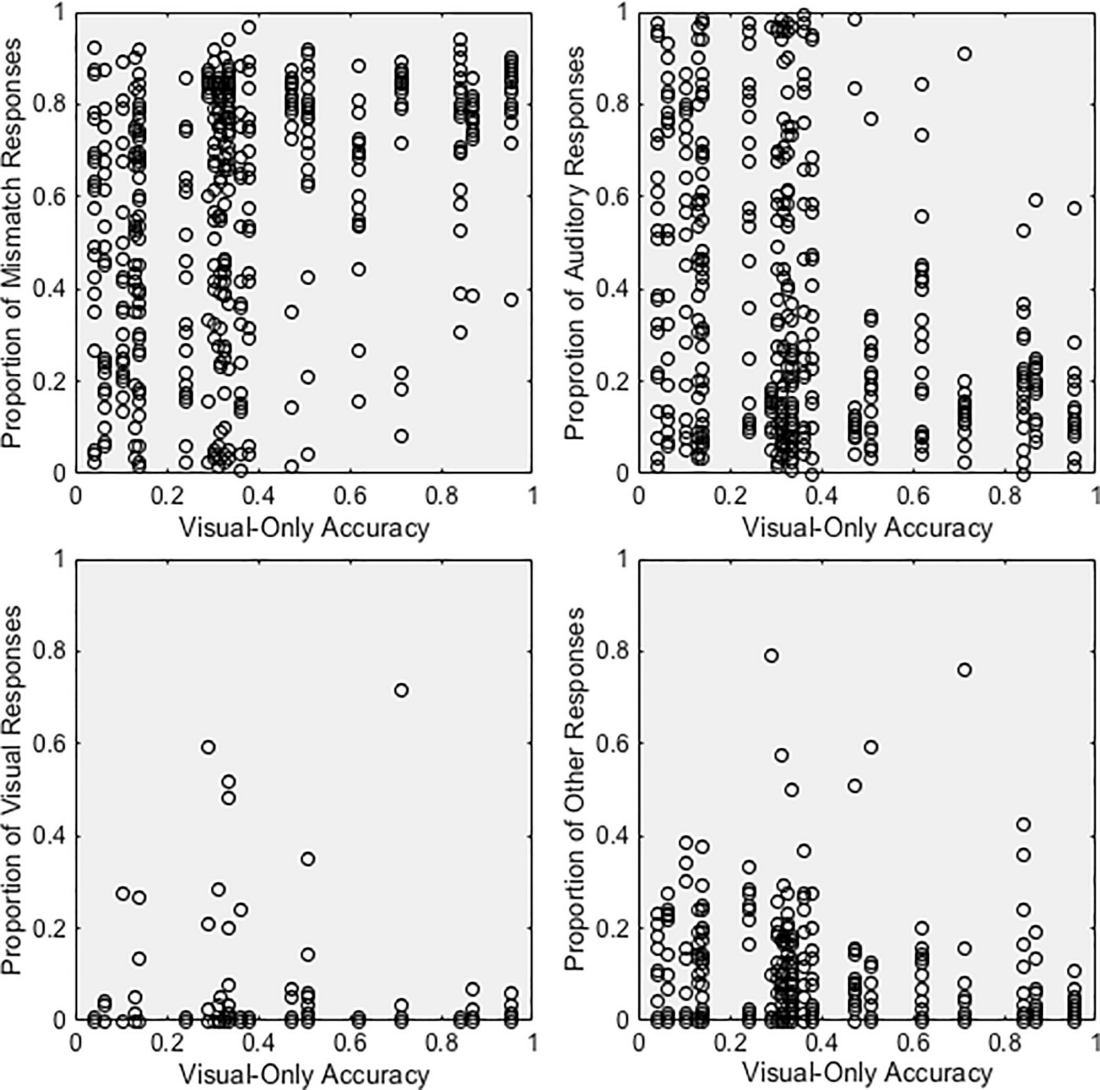
Responses as a function of visual-only accuracy. Proportion of (A) mismatch responses, (B) auditory responses, (C) visual responses, and (D) Other responses as a function of visual-only identification accuracy.

Previous research shows that the relative reliability of auditory and visual speech information determines how much each modality affects perception. One modality or cue has its greatest influence on perception when a second modality or cue is most ambiguous [11, 15]. Therefore, we would expect to see greater visual influence for auditory consonants with low auditory accuracy and greater auditory influence for visual consonants with low visual accuracy. Intuitively, we might also expect more mismatch responses when the speech information is reliable (and non-redundant) in both modalities. To test this notion, we examined the relationship between unimodal accuracy and responses to incongruent stimuli. Specifically, we used Kendall’s tau to examine the relationship between the proportion of mismatch, auditory, visual, and Other responses for consonants and auditory-only (Fig 5) and visual-only accuracy (Fig 6). Results are discussed for each response type below.

#### Congruency of speech features

**Fig 7:**
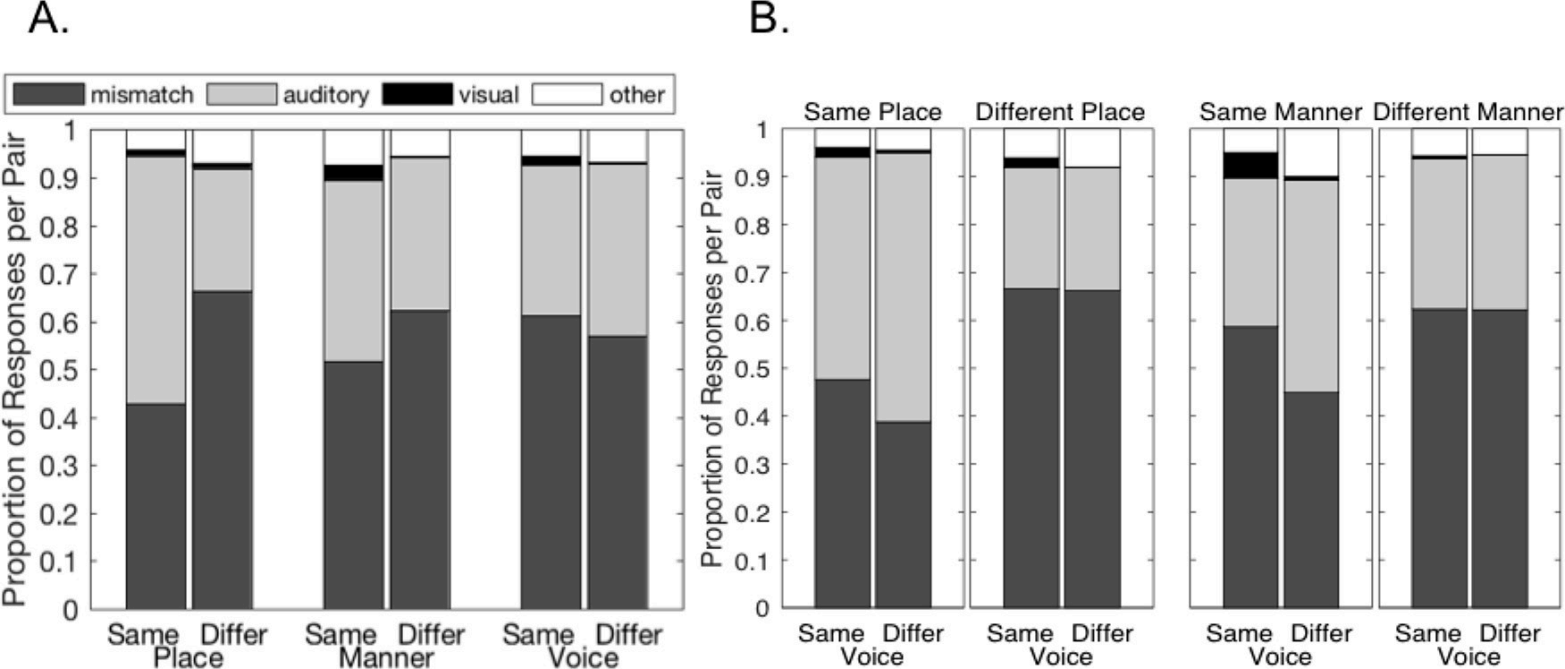
Responses to same-feature and different-feature incongruent AV consonant pairs. (A) Proportion of each response type as a function of whether auditory and visual consonant features (place, voice, manner) were the same or different. S2 Fig shows means and standard deviations. (B) Interaction of voice and place and voice and manner shows that some voice effects are an artefact of the interdependence of speech features.

Previous literature suggests that the perception of incongruent auditory and visual consonants depends on which articulatory features differ across modalities [10–12]. MacDonald and McGurk [14] hypothesized that perception of incongruent AV speech is determined by visual place and auditory voicing and manner (manner-place hypothesis). Additionally, perception of congruent AV consonants is primarily determined by visual place information and auditory voicing and manner information [4, 22, 23]. To examine how speech feature congruency relates to causal inference and incongruent AV speech perception, we examined whether responses to incongruent AV speech depend on a match in place of articulation, manner of articulation, and voicing between the auditory and visual consonants. Feature assignment for each consonant is shown in Table 2. We used a front-middle-back distinction for place of articulation [16, 24]. To avoid conflating voicing with other features, only consonants with voiced and voiceless cognates were included in the voicing analysis. Nasals, approximants, and the fricative /h/ were excluded. Paired-samples t-tests were used to compare same-feature and different-feature pairs. Results are show in Fig 7 and discussed for each response type below.

**Table 2:** Feature assignment for each consonant.

Feature	p	t	k	f	θ	s	ʃ	b	d	g	v	ð	z	ʒ	m	n	w	r	j	l	h	tʃ	dʒ
Place	0	1	2	0	1	1	2	0	1	2	0	1	1	2	0	1	0	1	2	1	2	2	2
Manner	0	0	0	1	1	1	1	0	0	0	1	1	1	1	2	2	3	3	3	3	1	1	1
Voicing	0	0	0	0	0	0	0	1	1	1	1	1	1	1	1	1	1	1	1	1	0	0	1

*Note*. Manner: 1 = stop, 2 = fricative, 3 = approximant, 4 = nasal; Place: 0 = front, 1 = middle, 2 = back.

#### Place of articulation

**Fig 8:**
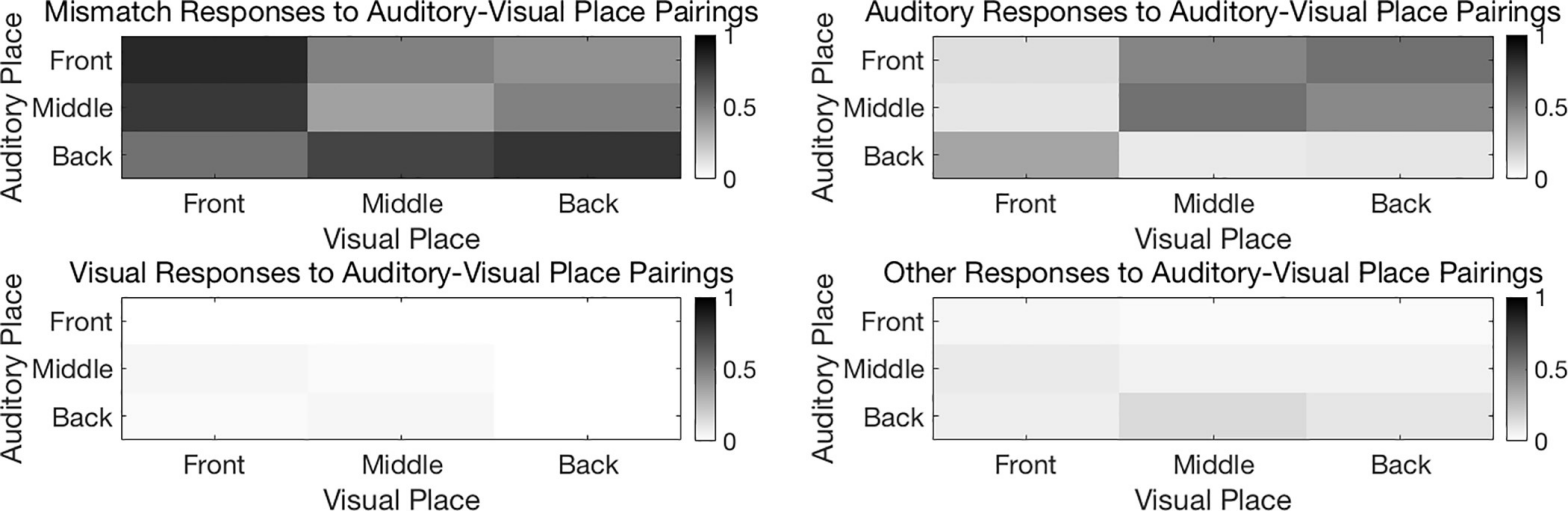
Responses as a function of place of articulation. Proportion of (A) mismatch responses, (B) auditory responses, (C) visual responses, and (D) Other responses as a function of auditory and visual place of articulation. See S3 Fig for means and standard deviations.

The visual signal conveys information about place of articulation better than it conveys other speech features [24], and front place of articulation is easier to see. Therefore, we expect to observe more visual influence for visual front consonants. However, given the high accuracy of unimodal auditory consonant identification, we may also expect participants to be more aware of the mismatch between auditory and visual information for front visual consonants. Two-way repeated measures ANOVAs were used to further explore effects of auditory and visual place of articulation on mismatch, auditory, visual, and Other responses. One-way ANOVAs were used to explore significant interactions, and paired-samples t-tests with Bonferroni corrections for multiple comparisons were used to explore significant effects (critical *p* = 0.0167). Results are shown in Fig 8 and discussed for each response type below.

### Mismatch responses

For the majority (59%) of incongruent AV consonant pairs in this experiment, participants noticed that the auditory and visual signals did not match (Fig 3A). These consonant pairs violated participants’ expectations about what visual and auditory features should go together, and so they did not integrate the incongruent signals. The results below indicate which distinctions between the auditory and visual speech signals dictated participants’ causal inference judgments.

#### Viseme clusters

Mismatch responses were more common when the auditory and visual consonants were visually distinct. Specifically mismatch responses were more than 3-times more common when the auditory and visual consonants belonged to different viseme categories, *t* = -7.9406; *p* < 0.0001; df = 7 (Fig 4).

#### Unimodal accuracy

Participants were more likely to notice the mismatch between consonants if visual consonant identification accuracy was high, r_τ_ = 0.2504, p < 0.0001. Fig 6A suggests that this relationship is driven by the high likelihood of a mismatch response for visual consonant identified with >40% accuracy. When the visual consonant was easier to identify, participants were less likely to integrate it with the auditory information.

#### Congruency of speech features

The congruency of place and manner information played an important role in participants’ causal inference judgements. Differences in place and manner made participants more likely to report a mismatch between auditory and visual consonants: place, t = -12.0579, p < 0.00001, df = 7, and manner, t = -8.814, p < 0.0001, df = 7. This was not the case for voicing. Participants were more likely to notice that the signals did not match when auditory and visual consonants had the same voicing, t = 6.6101, p = 0.0003, df = 7 (Fig 7A).

The finding that consonants that share a feature (voicing) are more likely to result in mismatch responses may initially seem contradictory. However, this voicing effect likely reflects the lack of independence among speech features: because all consonants differ in place, voice, or manner, consonants with the same voicing must differ in place and/or manner. To test the possibility that the voicing effect is simply an artefact, we conducted two-way repeated measures ANOVAs 1) with voice congruency and place congruency as independent variables and 2) with voice congruency and manner congruency as independent variables (Fig 7B). There were significant interactions of voice and place congruency, F = 30.0541, p = 0.00092, df = 1,7, and voice and manner congruency, F = 53.6955, p = 0.00016, df = 1,7. Post hoc comparisons demonstrated that voicing effects were only significant for same place and same manner pairs (p ≤ 0.0003), suggesting that the voicing effects are an artefact of the place and manner effects.

#### Place of articulation

On average, the proportion of mismatch responses increased with increasing distance between auditory and visual place of articulation (Fig 8A). For middle auditory and visual consonants, mismatch responses were also more common when paired with front consonants than with back consonants. Statistical analysis confirmed a significant interaction of auditory and visual place, *F*(4,28) = 72.31; *p* < 0.0001. Post hoc comparisons with Bonferroni correction for multiple comparisons consistently supported the stated trends (p ≤ 0.001), with one exception: There was no statistically significant difference between visual back/auditory middle consonant pairs and visual back/auditory back consonant pairs after Bonferroni corrections for multiple comparisons (p = 0.031).

#### Summary

In summary, mismatch responses occurred when consonants belonged to different viseme clusters and had different manners and places of articulation, especially when place of articulation was extremely different (front-back). Mismatch responses were more common for visual consonants associated with high visual accuracy. When participants could accurately identify visual consonants, they were more likely to notice that what they saw and heard did not match. In other words, these results suggest stimulus uncertainty governs causal inference in AV speech perception. Low visual stimulus uncertainty (high visual accuracy) decreases the likelihood of integrating incongruent auditory and visual signals. Although the same relationship was not observed for auditory consonant accuracy, this analysis was limited by the overall high accuracy of identification for most auditory consonants.

### Auditory responses

When participants were not aware that the AV syllables were incongruent, they reported the auditory consonants 82% of the time (Fig 3B). Each of the results detailed below suggest that auditory responses occurred when there was high redundancy between the auditory and visual speech signals.

#### Viseme clusters

Auditory responses occurred more often when the auditory and visual consonants were not visually distinct. Specifically, auditory responses were more common for auditory and visual consonants that belonged to the same viseme cluster, *t* = 10.9263; *p* < 0.0001, df = 7 (Fig 4).

#### Unimodal accuracy

Auditory responses were also more common for auditory consonants with high auditory accuracy, r_τ_ = 0.2702, p < 0.0001 (Fig 5B). Participants were nearly twice as likely to report the auditory consonant if the auditory stimulus was identified with 100% accuracy in auditory-only conditions than if it was identified with less than 100% accuracy. Conversely, auditory responses were more common for visual consonants with low visual accuracy, r_τ_ = -0.1786, p < 0.0001. Fig 6B suggests that this relationship is driven by the low likelihood of an auditory response for visual consonant identified with >40% accuracy. These results are consistent with the notion that auditory cues have a greater influence on perception when visual cues are more ambiguous [11, 15].

#### Congruency of speech features

Auditory responses occurred more for incongruent consonant pairs with the same place, t = 10.876, p < 0.0001, df = 7, and manner, t = 5.6223, p = 0.0008, df = 7 of articulation and for incongruent consonant pairs differing in voicing, t = -7.6744, p = 0.0001, df = 7 (Fig 7A). A comparison of cognates in Fig 3B suggests that this voicing effect may be driven by consonants differing only in voicing. If the only difference between the auditory and visual consonants was voicing (a feature that is not well-represented by the visual signal), participants were willing to accept that it matched the auditory consonant. This is consistent with previous findings (e.g., [14]).

As with mismatch responses, we tested whether the voicing effects were an artefact of the relationship between congruency of the voice feature and congruency of the manner and place features. Two-way repeated measures ANOVAs with voice congruency and place congruency as independent variables and with voice congruency and manner congruency as independent variables revealed significant interactions of voice and place, F = 46.0899, p = 0.00025, df = 1,7 and voice and manner, F = 32.4448, p = 0.00073, df = 1,7. Once again, post hoc comparisons demonstrated that voicing effects were only significant for same place and same manner pairs, p ≤ 0.0002, suggesting that the voicing effects are an artefact of the place and manner effects (Fig 7B).

#### Place of articulation

Auditory responses were more common when consonants had the same place of articulation. When place of articulation differed across consonants, auditory responses were more common with more back articulations. Fig 8B shows a significant interaction of auditory and visual place, *F*(4,28) = 72.311; p < 0.0001. Post hoc comparisons with Bonferroni corrections for multiple comparisons generally supported the trends noted (p ≤ 0.013) with one exception: There was no significant difference between visual front/auditory middle and visual front/auditory back consonants after correcting for multiple comparisons (p = 0.03).

#### Summary

In summary, auditory responses occurred when there was low disparity between the auditory and visual consonants (when they belonged to the same viseme cluster, when they had the same manner and place of articulation) and when there was greater ambiguity in the visual information (when the visual consonant was poorly identified and when consonants differing in place of articulation were produced further back).

### Visual responses

Visual responses were uncommon, accounting for less than 2% of responses to incongruent stimuli (Fig 3C). Table 3 shows the incongruent consonant pairs for which at least 10% of responses were consistent with the visual syllable, the percentage of visual responses to each consonant pair, and whether the place, voice, manner, and viseme cluster differed across the auditory and visual consonants. Visual responses were associated with auditory-only errors. For example, if a unimodal auditory-only /v/ was commonly mistaken for /b/ (Fig 1B), then the incongruent auditory /v/–visual /b/ consonant pair sometimes resulted in a visual response. The percentage of corresponding substitutions from unimodal auditory testing are shown in the last column of Table 3. This pattern was consistent; the consonant pairs in Table 3 account for 93% of auditory-only consonant confusions. Given their relationship to auditory errors, one interpretation of these visual responses is that they are simply auditory responses with errors. However, in most cases, the percentage of visual responses is higher than the percentage of unimodal auditory substitutions, suggesting that the incongruent visual consonant truly influenced responses. The exception to the relationship between unimodal auditory errors and visual responses to incongruent stimuli is the fricative /h/. The /h/ consonant was identified perfectly in auditory-only conditions, but resulted in a visual response when paired with dental and post-alveolar fricatives and affricates. The visual consonants clearly influenced the perception of the auditory /h/.

**Table 3:** Incongruent consonant pairs resulting in visual responses.

Auditory Consonant	Visual Consonant	% Visual Responses	Place	Voice	Manner	Viseme Cluster	% Unimodal Auditory Substitution
θ	f	72%	Differ	V-	Fricative	Differ	21%
f	θ	52%	Differ	V-	Fricative	Differ	9%
ð	v	59%	Differ	V+	Fricative	Differ	10%
v	ð	34%	Differ	V+	Fricative	Differ	8%
ð	z	13%	Differ*	V+	Fricative	Differ	6%
θ	s	24%	Differ*	V-	Fricative	Differ	5%
f	ð	14%	Differ	Differ	Fricative	Differ	5%
ð	θ	20%	Interdental	Differ	Fricative	Differ	17%
θ	ð	14%	Interdental	Differ	Fricative	Differ	7%
h	ʃ	25%	Differ	V-	Fricative	Differ	0%
h	θ	21%	Differ	V-	Fricative	Differ	0%
h	ʒ	31%	Differ*	Differ	Fricative	Differ	0%
h	ð	19%	Differ	Differ	Fricative	Differ	0%
h	tʃ	28%	Differ*	V-	Differ	Differ	0%
v	b	48%	Differ*	V+	Differ	Differ	38%
b	v	21%	Differ*	V+	Differ	Differ	1%
ʒ	dʒ	23%	Alveolar	V+	Differ	Same	32%
n	m	28%	Differ	V+	Nasal	Differ	2%
m	n	28%	Differ	V+	Nasal	Differ	5%

*Note*. Only contrasts for which at least 10% of responses were consistent with the visual syllable are included in the table.

*Denotes that this contrast would be considered the same place of articulation using the front-middle-back distinction [16, 24].

#### Viseme clusters

The consonant pairs with the highest portions of visual responses mostly belonged to different viseme clusters (Table 3). Statistical analysis confirmed that visual responses occurred more often when the auditory and visual consonants belonged to different viseme categories, t = -2.8933, p = 0.023, df = 7. (Fig 4).

#### Unimodal accuracy

Although the relationship was rather weak, visual responses occurred more for auditory consonants with lower unimodal auditory identification accuracy, r_τ_ = -0.1017, p = 0.0093, Fig 5C, and visual consonants with lower visual identification accuracy, r_τ_− 0.1019, p = 0.0049, Fig 6C. However, note that these findings are likely strongly influenced by the few consonant pairs in Table 3 with >10% visual responses.

#### Congruency of speech features

Visual responses occurred more for incongruent consonants with the same manner, t = 6.8422, p = 0.0002, df = 7, and voicing, t = 5.5476, p = 0.00086 (Fig 7A). Unlike mismatch and auditory responses, the effect of voicing on visual responses does not appear to be an artefact of the relationship between voicing and manner/place features. There were significant voice congruency effects for same place, same manner, different place, and different manner pairs, p ≤ 0.0014 (Fig 7B).

There was no significant difference in visual responses for same place and different place pairs, at least for the relatively broad front-middle-back distinction used in this investigation. However, note the slight (within place category) differences in place of articulation for many of the consonant pairs in Table 3.

#### Place of articulation

Visual responses were less common if either consonant was produced in the back of the mouth, and did not depend on whether place was the same or different across consonants (at least using the relatively broad front-middle-back distinctions). Fig 8C shows a significant interaction of auditory and visual place, *F*(4,28) = 23.3724; *p* < 0.0001. The effect of place was only significant for front and back auditory and visual consonants, F(2,14) ≥ 26.610, p < 0.001. Post hoc comparisons indicated that visual responses were more common when not paired with a back consonant, p ≤ 0.003. Additionally, participants reported the visual syllable more for middle consonants paired with front consonants than for middle consonants paired with middle consonants, p ≤ 0.014.

#### Summary

In summary, visual responses were associated with auditory-only errors. However, the proportions of visual responses were higher than would be expected if they simply reflected auditory responses with errors. This suggests that, despite the large overall differences between auditory and visual stimulus ambiguity, visual speech influenced perception of some integrated incongruent AV consonant pairs. This visual influence was more prominent for front and middle consonants than back consonants, and with consonants sharing the same voice and manner.

### Other responses

An Other response represents a time when a participant did not notice the mismatch between the auditory and visual consonants, and instead combined some components of each modality into an integrated percept. There were a wide variety of “Other” responses. Fig 3D shows the proportion of Other responses as a function of auditory and visual consonant. For a few consonant pairs, the main response was Other. To clarify the nature of the Other responses, Table 4 shows the incongruent consonant pairs for which the majority of responses were Other. Additionally, Table 5 summarizes the types of Other responses.

**Table 4:** Most common other responses to incongruent consonant pairs.

Auditory Consonant	Visual Consonant	Dominant Response	Number of Responses	Place	Voice	Manner
θ	v	f	86/95	V	A	Both
ð	f	v	84/91	V	A	Both
f	ð	θ	70/71	V	A	Both
v	θ	ð	56/60	V	A	Both
ð	s	z	33/44	V	A	Both
v	m	b	67/69	V	Both	Neither
v	p	b	57/61	V	A	V

*Note*. This table only includes consonant pairs for which the primary response was “other.”

**Table 5:** Summary of other responses.

Nature of Response			% “Other”
Change in only place re: auditory consonant			49.1%
auditory fricatives	70.3%	(7.8% per consonant)	
auditory nasals	17.7%	(8.9% per consonant)	
auditory stops	11.7%	(2.0% per consonant)	
auditory approximants	0.3%	(< 0.1% per consonant)	
Consonant cluster containing (features of) both consonants			15.3%
/m[C]*/ with visual /p, b, m/		61.4%	
/sθ/ with auditory /θ, f/		17.3%	
/w[C]*/ with visual /w/		4.3%	
Auditory /bi/ and /bu/ changed to /i/ and /u/			4.7%
front visual consonant	5.1%	(1.5% per consonant)	
middle visual consonant	39.6%	(7.8% per consonant)	
back visual consonant	55.4%	(10.9% per consonant)	
Change in only manner re: auditory consonant			12.5%
/dʒ/ for auditory /ʒ/		58.6%	
/b/ for auditory /v/		33.0%	
/v/ for auditory /b/		4.9%	
Change in only voice re: auditory consonant			10.1%
/ð/ for auditory /θ/		49.7%	
/θ/ for auditory /ð/		46.8%	
Fricatives that change voice and place re: auditory consonant			3.8%
/θ/ for auditory /v/		79.0%	
/ð/ for auditory /f/		21.0%	
Nature of Response			% “Other”
Change in only place re: auditory consonant			49.1%
auditory fricatives	70.3%	(7.8% per consonant)	
auditory nasals	17.7%	(8.9% per consonant)	
auditory stops	11.7%	(2.0% per consonant)	
auditory approximants	0.3%	(< 0.1% per consonant)	
Consonant cluster containing (features of) both consonants			15.3%
/m[C]*/ with visual /p, b, m/		61.4%	
/sθ/ with auditory /θ, f/		17.3%	
/w[C]*/ with visual /w/		4.3%	
Auditory /bi/ and /bu/ changed to /i/ and /u/			4.7%
front visual consonant	5.1%	(1.5% per consonant)	
middle visual consonant	39.6%	(7.8% per consonant)	
back visual consonant	55.4%	(10.9% per consonant)	
Change in only manner re: auditory consonant			12.5%
/dʒ/ for auditory /ʒ/		58.6%	
/b/ for auditory /v/		33.0%	
/v/ for auditory /b/		4.9%	
Change in only voice re: auditory consonant			10.1%
/ð/ for auditory /θ/		49.7%	
/θ/ for auditory /ð/		46.8%	
Fricatives that change voice and place re: auditory consonant			3.8%
/θ/ for auditory /v/		79.0%	
/ð/ for auditory /f/		21.0%	

[C]* represents the auditory consonant or (infrequently) a consonant sharing features with the auditory consonant.

The consonant pairs resulting in majority Other responses include several fricative-fricative pairs differing in both place and voicing. As the manner-place hypothesis [14] would predict, the response to these fricative-fricative pairs matched the place of the visual stimulus and the voicing of the auditory stimulus. Other was also the majority response for auditory /v/ paired with visual /p/ and /m/ (response /b/). This result can readily be explained in terms of three observations: 1) auditory /v/ was commonly mistaken for /b/; 2) auditory /v/ paired with visual /b/ resulted in a visual /b/ response; and 3) /p/, /b/, and /m/ are visually confusable. These particular Other responses may therefore reflect either an auditory response with an error or visual influence.

Although these were the only incongruent consonant pairs for which the majority of responses were Other, additional patterns emerged (Table 5). Notably and consistent with the manner-place hypothesis [14], 49.1% of Other responses matched the manner and voice of the auditory signal, and the place of the visual signal. Another 15.3% were consonant clusters with consonants matching and/or sharing features with both the auditory and visual signals. Most of these were /m[C]*/ blends (see Table 5), resulting when visual /p, b, m/ was paired with an auditory consonant from a different viseme category (i.e., reporting /mtɑ/ for auditory /m/—visual /t/). Finally, 4.7% of Other responses were reports of only the vowel when auditory /bi/ and /bu/ consonants were paired with middle and back visual consonants. The remaining common patterns were related to auditory-only errors (i.e., responding /ð/ for auditory /θ, f/ and responding /b/ for auditory /v/).

#### Viseme clusters

Although the most common Other responses were for auditory and visual consonant from different viseme clusters (Table 4), the difference between the proportion of Other response for within- and between-viseme-cluster pairs failed to reach significance, t = -2.23–1, p = 0.061 (Fig 4).

#### Unimodal accuracy

Other responses were more common for auditory consonants with lower levels of auditory accuracy, r_τ_ = -0.5299, p < 0.0001 (Fig 5D). The proportion of Other responses was not correlated with visual accuracy.

#### Congruency of speech features

Other responses occurred more for incongruent consonant pairs with the same manner, t = 4.2925, p = 0.0036, df = 7, and for incongruent consonant pairs differing in voicing, t = -4.9845, p = 0.0016 (Fig 7A). In this case, voice congruency interacted with place congruency, F = 23.7356, p = 0.0018, df = 1,7, and manner congruency, F = 63.1883, p = 0.00009, df = 1,7. Post hoc comparisons indicated that the voicing effect was significant for different place pairs, t = -6.5283, p = 0.00033, df = 7 and same manner pairs, t = -7.1821, p = 0.00018, df = 7 (Fig 7B). This is consistent with the common pairs in Table 4, which mostly have different voice, different place, and same manner.

#### Place of articulation

There is a significant effect of auditory place of articulation, F = 6.4668, p = 0.0103, df = 2, 14, and a significant interaction of auditory and visual place of articulation on Other responses, F = 2.7865, p = 0.0457, df = 4, 28 (Fig 8D). In general, front-middle consonant pairs were more likely to result in Other responses than any other consonant pairs. After Bonferroni corrections, post hoc comparisons indicated that auditory front-visual middle consonant pairs resulted in more Other responses than auditory front-visual front and auditory front-visual back consonant pairs, and visual front-auditory middle consonant pairs resulted in more Other responses than visual front-auditory back consonant pairs (p ≤ 0.014).

#### Summary

In summary, Other responses were highly variable. When the incongruent consonants differed in place, the response was more likely to match the visual place. When the two consonants differed in voice or differed in manner, the response tended to match the auditory voice and the auditory manner. This finding is consistent with MacDonald and McGurk’s [14] manner place hypothesis and with previous studies demonstrating that congruent AV consonant perception is primarily determined by visual place information and auditory voicing and manner information [4, 22, 23]. This finding likely reflects the relatively high salience of place information in the visual signal (compared to visual manner and voicing information) and relatively low salience of place information in the auditory signal (compared to auditory manner and voicing information) [24].

## Modeling AV speech from unimodal responses

Most models of AV speech integration have yet to incorporate causal inference [8 for an exception]. We expect the data from the current study will be useful when researchers begin to do so. To demonstrate, we modified a fixed (parameter-free) version of the FLMP [10, 15, 25] to incorporate causal inference judgements. We used the modified FLMP to predict response to AV speech (including mismatch detection) based on confusions among unimodal auditory and unimodal visual consonants.

In the FLMP, stimulus information in each modality is evaluated independently to determine the degree to which it matches various response alternatives/prototypes in memory. The degree of support for each alternative/prototype is estimated from unimodal confusions. Then, the degree of support from each modality is combined to provide an overall degree of support for each prototype or response alternative [15]. During the decision stage, the goodness of match between the stimulus and prototype is evaluated relative to the summed goodness of match of the other prototypes [25]. For example, the support for a /bɑ/ response/prototype is given by the product of the degree to which the auditory /bɑ/ stimulus supports /bɑ/ (*a*_*ba*|*ba*_) and the degree to which the visual /bɑ/ stimulus supports /bɑ/ (*v*_*ba*|*ba*_), relative to the support for all other response alternatives *s*(*r*). The response alternative with the highest relative support is the model response.

$$s(/ba/)=\frac{a_{ba|ba}v_{ba|ba}}{\sum_{r}s(r)}$$ (1)

In the FLMP, the stimulus information in one modality does not change the evaluation of stimulus information in the other modality. To model the perception of incongruent speech, we assume that participants only have congruent speech prototypes. Therefore, the degree to which each incongruent AV speech signal supports each prototype/response alternative can also be estimated from unimodal confusions. Using the same equation, we can calculate the degree of support for a particular response to an incongruent AV stimulus. For example, the support for a /bɑ/ response to an incongruent auditory /dɑ/—visual /mɑ/ stimulus would be calculated as:

$$s(/ba/)=\frac{a_{ba|da}v_{ba|ma}}{\sum_{r}s(r)}$$ (2)

where *a*_*ba*|*da*_ represents the auditory support for response /bɑ/ given auditory /dɑ/, and *v*_*ba*|*ma*_ represents the visual support for response /bɑ/ given visual /mɑ/.

To evaluate FLMP prediction of incongruent AV consonant perception, we used the participant- and vowel-specific unimodal confusion matrices (shown in S4 Fig and averaged across vowel contexts in Fig 1A and 1B) to calculate unimodal support for each response alternative, given each (congruent and incongruent) AV stimulus. To incorporate causal inference, we made one change to the model: Note that support values cannot be calculated if the support for all responses is 0 (if ∑_*r*_ *s*(*r* = 0). This would occur if no response alternative was supported to some degree by *both* the auditory stimulus and the visual stimulus. When ∑_*r*_ *s*(*r* = 0) for a given AV stimulus, we marked the model response as a mismatch response. Thus, in the model, when there is no response alternative that is supported by both the auditory and the visual information, the model reports being aware of the mismatch between the auditory and visual signals.

**Fig 9:**
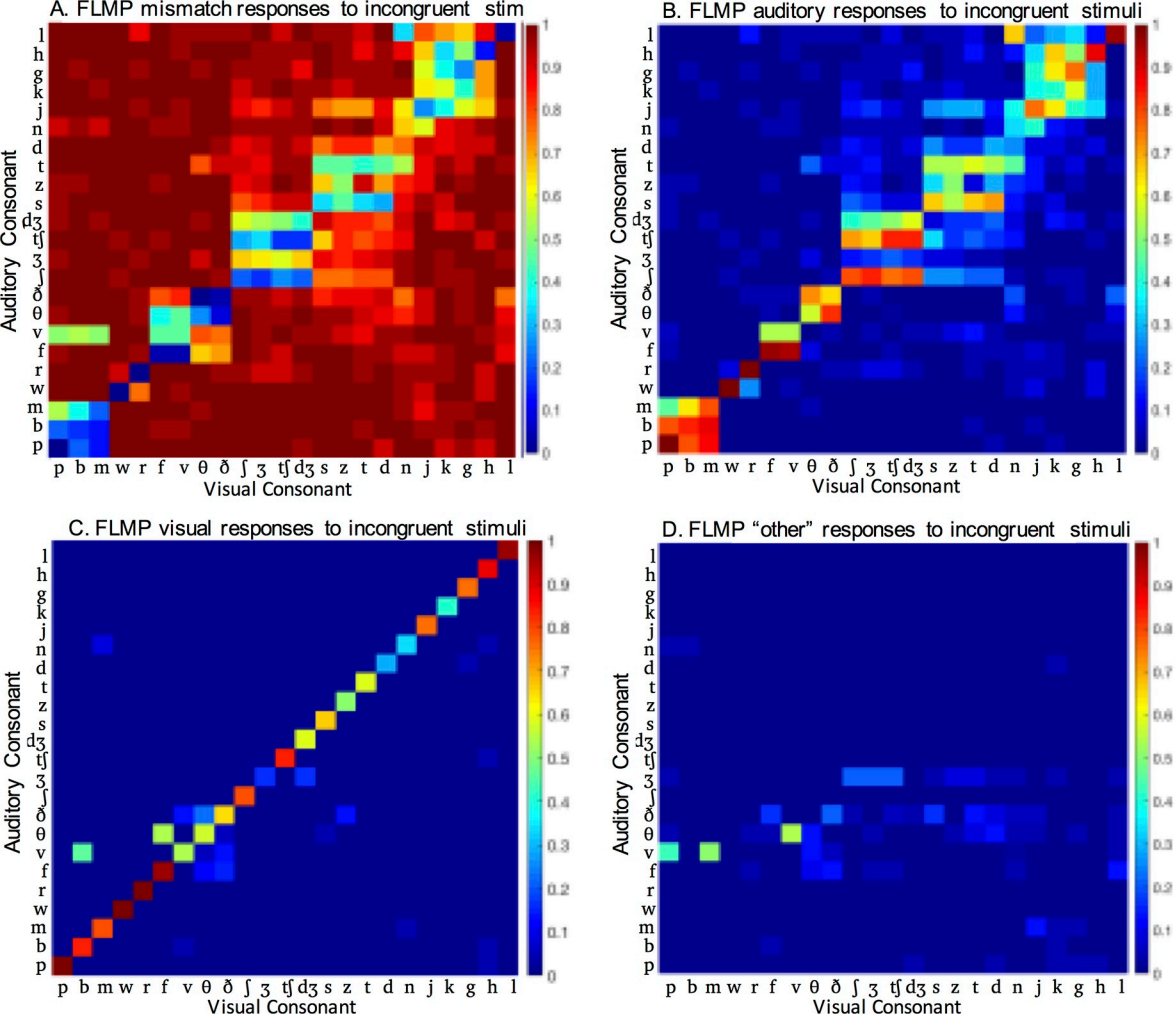
Modeled responses to incongruent AV consonants. FLMP modeled proportion of (A) mismatch responses, (B) auditory responses, (C) visual responses, and (D) Other responses to AV syllables, as a function of auditory and visual consonant.

**Fig 10:**
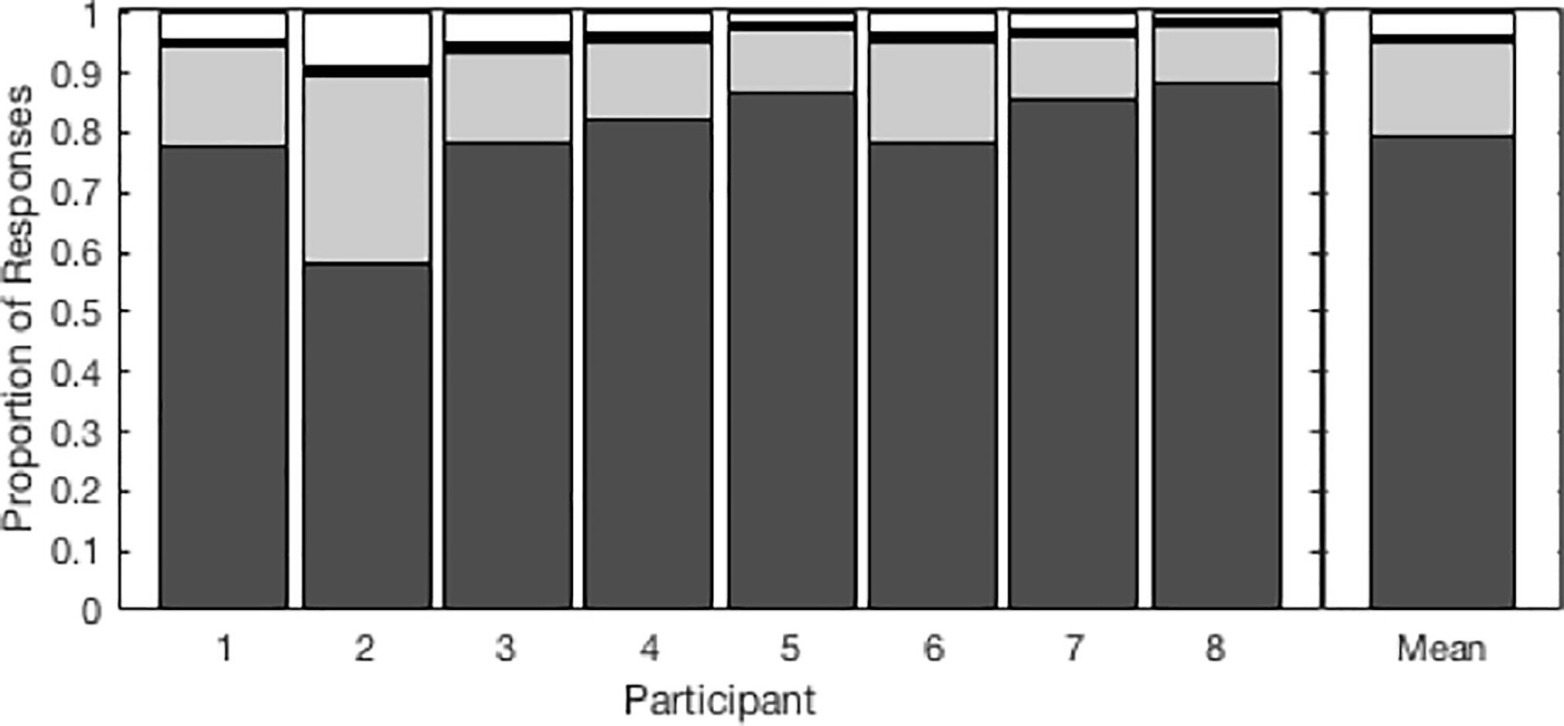
Modeled proportions of each response type for incongruent AV syllables. FLMP modeled individual and mean proportion of mismatch, auditory, visual, and Other responses to incongruent syllables.

Modeled AV responses are shown in Fig 9. Model responses are qualitatively similar to behavioural responses in Fig 3. Notably, the same effects of viseme category are visible in the modeled and behavioral data. However, the model overestimated the proportion of mismatch responses by about 28% (Fig 10, as compared to Fig 2). The model also underestimated the proportion of correct responses to congruent AV consonants mixed with incongruent AV consonants. Interestingly, it captured individual differences in the proportion of each response type quite well (Fig 10, as compared to Fig 2).

To evaluate the goodness of fit of the model data, we calculated the RMSD (root mean squared difference) between the modeled data and behavioral data. For comparison, we calculated RMSD for both the congruent AV (Fig 1B) and incongruent AV (Fig 3) data sets. The RMSD for the congruent stimuli was 0.1557. The fit for the incongruent stimuli was just as good as the congruent stimuli (RMSD = 0.1489). These results confirm that—with a causal inference rule—the parameter-free version of the FLMP can predict responses to incongruent AV stimuli based on unimodal confusion matrices as well as it predicts responses to congruent AV stimuli based on the same confusion matrices.

## Discussion

Traditional research on AV speech perception typically assumes that participants will integrate incongruent auditory and visual speech signals. However, we often are able to detect when auditory and visual speech does not match. Causal inference—the process of deciding whether incoming auditory and visual signals come from the same source—is an important step in AV speech perception [8]. The purpose of this investigation was to explore the rules governing causal inference in perception of incongruent AV English consonants and the pattern of perceptual responses that occurs after controlling for causal inference. For the majority of incongruent AV consonant pairs (59%), participants were aware of the mismatch between the auditory and visual consonants, highlighting the need to incorporate causal inference as a key step in AV speech perception models [8].

We analyzed causal inference judgments and patterns of incongruent AV consonant confusions, and related them to 1) unimodal accuracy, 2) auditory and visual place of articulation, 3) match/mismatch of auditory and visual articulatory features, and 4) patterns of unimodal consonant confusions. Overall, causal inference and perception of incongruent AV consonants depended on 1) the salience and reliability of the auditory and visual features/inputs and 2) the degree of redundancy between the auditory and visual inputs. The data from the current study present an opportunity to test and potentially improve the generalizability of current AV speech integration models, and will be valuable as researchers begin to incorporate causal inference into computational models of AV speech perception.

### Causal inference

For nearly 60% of incongruent trials, participants were aware of the mismatch between the auditory and visual consonants. As noted, participants were aware of the mismatch when there was salient information in both modalities (high auditory and visual accuracy) with low redundancy between the auditory and visual consonants (different place, different manner, and different viseme categories). These results suggest that salience, redundancy, and stimulus certainty guide decisions about causal interference.

This finding diverges from those of Jiang and Bernstein [8], who found that low physical correspondence was associated with fusion responses. Jiang and Bernstein did not give participants the option of reporting that the stimuli did not match. In fact, most participants in that study reported—at the end of the experiment—that they were sometimes aware that the consonants did not match. These results suggest that an open-set response may be necessary to truly capture how participants perceive incongruent AV speech.

The finding that participants were typically aware of the mismatch between the auditory and visual consonants has implications for computational models of AV speech integration. These models typically compute a response, regardless of the disparity between the auditory and visual signals (i.e., [8, 10, 26]). Even in Magnotti and Beauchamp’s model of causal inference in multisensory speech perception (CIMS) [21], a response is computed for each trial. The results of the current study suggest that models include a computational rule for determining when participants will fail to integrate the auditory and visual signals. In fact, by incorporating a simple rule for detecting mismatch into the FLMP [15], we were able to model responses to incongruent AV speech as well as we were able to model responses to congruent AV speech.

Awareness of the incongruence between auditory and visual consonants could result because participants detected a mismatch between amodal physical features of the auditory and visual stimuli (i.e., onsets, duration, amplitude envelope) [27, 28]. It could also reflect a violation of experience-based knowledge about which speech sounds and facial gestures are associated with one another. The finding that visual salience (front place) of the *auditory* consonant was an important determinant of mismatch responses suggests that experience-based knowledge plays a role. As noted, participants were more likely to report being aware that consonants were incongruent when either consonant was produced in the front of the mouth. Whereas front *visual* consonants features are easier to see than back visual consonants, front *auditory* consonant features are no clearer than middle/back auditory consonant features. The fact that participants also noticed the mismatch more with front auditory consonants suggests that their expectations about what visual features would accompany the auditory consonant were violated more often for front auditory consonants. It seems that participants had more precise expectations about what visual features should accompany front auditory consonants. Whereas they many have been willing to accept that *any* consonant without a non-labial closure matched a middle or back auditory consonant, they may have had more precise expectations about the onset, location, duration, and/or degree of closure of front consonants.

We should note that in the current study, mismatch responses were far more common than fused responses for traditional McGurk illusion stimuli. Several aspects of the methodology in the current study likely contributed to the high proportion of mismatch responses and to this discrepancy between the current and previous findings. First, simply offering participants the option to report when they were aware that the auditory and visual consonants did not match may have created a bias that encouraged participants to search for mismatches between the auditory and visual stimuli. Additionally, the 22:1 ratio between incongruent and congruent AV trials may have biased participants to perceive the mismatch. Evidence for this bias is found in the fact that participants reported a mismatch for approximately 4% of congruent AV trials, when those trials were mixed with the incongruent AV trials. Second, the auditory stimuli were presented monaurally to the right ear, which introduced spatial disparity between the auditory and visual signals. The effects of spatial disparity were likely negligible for congruent AV speech and even some incongruent AV speech, given strong evidence that temporal congruency of auditory and visual information trumps spatial disparity [29–32]. All of the incongruent AV signals had simultaneous onsets, but it is possible that natural variation in the temporal characteristics of the auditory and visual signals interacted with spatial disparity to increase the proportion of mismatch responses.

We created incongruent AV stimuli by matching the audio and video portions based on the onset of the sound. This preserved the temporal relationship between the onset of the mouth movement and onset of the sound from the *visual component* of the incongruent AV syllable. Given the relatively high levels of unimodal auditory accuracy, the proportion of mismatch responses may have been lower (and the proportion of McGurk fusion responses higher) if we instead matched the audio and video portions based on the onset of the mouth movement, preserving the relationship between the onset of the mouth movement and onset of the sound from the *auditory component* of the incongruent AV syllable. However, previous work suggests that the same behavioural results are obtained whether speech is aligned based on the onset of the consonant or the onset of the vowel [9].These issues can be resolved in future studies, by examining patterns of perceptual confusions among incongruent AV consonants as a function of spatial and temporal disparity and the ratio of congruent to incongruent trials.

### Unimodal reliability

Because the stimuli in the current investigation were presented in quiet, participants identified the auditory signal with far greater accuracy than the visual signal (93% vs. 38% accuracy). Unimodal reliability appeared to determine responses to incongruent trials. Auditory responses to incongruent trials were far more common than visual responses to incongruent trials (34% vs. <2% of responses). In addition, auditory responses were higher for visual consonants with low visual-only identification, and visual responses occurred primarily when there was ambiguity in the auditory consonant.

These results are consistent with previous findings suggesting that visual speech is more influential when auditory information is neutral or ambiguous; and auditory speech is more influential when visual information is neutral or ambiguous. For example, visual speech affects /bɑ/ vs. /dɑ/ categorization more when the auditory signal is acoustically between /bɑ/ and /dɑ/ prototypes than when the auditory speech matches a /bɑ/ or /dɑ/ prototype [10]. Additionally, visual speech improves auditory speech processing more when the auditory signal is masked by background noise [10, 33]. Such effects of stimulus uncertainty are built into Bayesian models of AV speech integration [8, 12, 34]. Future studies should decrease the reliability of the auditory signal using auditory filtering or masking, and determine whether results generalize across changes in relative unimodal accuracy.

### Interaction of salience and redundancy

The results of the current investigation are largely explained by an interaction of auditory and visual stimulus salience and auditory and visual stimulus redundancy. When there was salient information in both modalities with low redundancy (i.e., when unimodal accuracy was high and consonants were visually distinct), participants were aware of the mismatch between the auditory and visual consonants. When there was high redundancy between the auditory and visual information (same viseme cluster, same manner, same place), participants tended to report the more reliable (auditory) consonant. These results are consistent with Jiang and Bernstein [8], who found auditory responses when there was high physical correlation between the auditory and visual signals. Participants also reported the auditory consonant when non-redundant cues had low salience (i.e., consonants differing in place that were not produced in the front of the mouth).

The small portion of visual responses occurred when the non-redundant auditory information had low salience. The internal representations activated by the visual consonant were difficult to distinguish auditorily from the presented auditory consonant. In other words, if the auditory consonant was confused for the visual consonant during auditory-only testing (high uncertainty, high redundancy), the incongruent AV pair was more likely to result in a visual response. Visual responses were also more common when the consonant place was more visually salient (i.e., more front). As in previous research from Jiang and Bernstein [8], back visual consonants were less visually salient and therefore weakly influenced perception of the auditory consonant.

The interaction of salience and redundancy arises in other areas of AV speech perception research. For example, salience and redundancy are central to the analysis-by-synthesis framework, which was developed to explain how visual speech information influences timing properties of auditory speech-evoked, event-related potentials [21]. Within this framework, the visual signal is used to predict the upcoming auditory information. The visual signal activates all compatible speech representations (i.e., all of the visemes of the signal). Then, the auditory signal is evaluated within the context of the viseme category. More salient visual signals—such as those produced at the front of the mouth—provide more precise predictions and thus bias perception of the auditory information more strongly [21]. In fact, front visual stops have been shown to shape perception of auditory stops in noise more than back visual stops [11]. Additionally, front visual stops result in greater temporal facilitation of ERP responses than back visual stops [21]. The salience of the unimodal signals interacts with the degree of redundancy between the signals. When there is low redundancy between the signals (i.e., the signals have different places of articulation), there is a large difference between the predicted auditory signal (based on the visual signal) and the perceived auditory signal. When there is high redundancy, the differences are small. Higher redundancy is associated with greater temporal facilitation of ERP responses [21].

### Redundancy and salience of articulatory features

Given the role of salience and redundancy, the current results indicate which features need to be redundant/non-redundant to result in a particular response. Whereas consonants with the same place or manner of articulation tended to result in auditory responses, consonants with different place or manner of articulation tended to result in awareness of a mismatch between the auditory and visual consonants. This suggests that redundancy of the place and manner features was important for forming a unified percept (although see previous McGurk illusion research [1]). Auditory responses were also more common when consonants differing in place did not include a front consonant. This suggests that—relative to middle and back consonants—we make more precise predictions about what auditory information accompanies front visual consonants and have more precise expectations about what visual information accompanies front auditory consonants.

Visual responses were highly uncommon when voice and manner differed. This suggests that redundancy of manner and voice information is important for visual responses and likely reflects the high salience of auditory voice and manner information. Place differences were the most susceptible to visual influence; The most common visual responses occurred when the consonants differed only in place of articulation (especially nasals /m, n/, fricatives /f, θ/ and /v, ð/, and auditory /h/ paired with other fricatives).

These results are consistent with previous demonstrations that AV consonant perception is primarily determined by visual place information and auditory voicing and manner information [4, 22, 23]. These results are also consistent with MacDonald and McGurk’s [14] hypothesis that the manner and voice of incongruent AV speech are determined by the auditory consonant, and the place of incongruent AV speech is determined by the visual consonant. “Other” responses were also consistent with this pattern: When place information conflicted, Other responses tended to match visual place. When voice or manner information conflicted, Other responses tended to match the auditory voice and auditory manner. Once again, we feel it important to note that results were not entirely consistent with MacDonald and McGurk’s hypothesis and data. The manner-place hypothesis does not include a role for causal inference and detecting mismatches, and would predict a much higher proportion of “Other” responses.

The current study used traditional definitions of articulatory features. The viseme categories derived from the visual-only data show discrepancies between the perceptually-based clusters and these classic speech features. Although these traditional categories are still highly relevant to our understanding of speech processing (e.g., [35]), the viseme categories and unimodal confusion matrices predicted performance better than these traditional features. Thus, as research begins to incorporate causal inference into models of AV speech perception, patterns of consonant confusions and perceptually-derived viseme clusters will likely prove more valuable than traditionally-defined articulatory features. The relations between the spectro-temporal characteristics of the auditory and visual signals [27, 28, 36] may also prove valuable.

### Individual variability

Although the data presented here represent nearly 64,000 trials, most were collected from only 8 participants. Additionally, there was some variability in the pattern of responses across participants, with two subjects showing lower proportions of mismatch responses than the other 6 participants. Despite these differences in the proportion of mismatch responses, these two subjects showed similar patterns with regard to the relationship between responses and the predictive variables.

Our modelling results provide some insight into why these two participants’ responses diverged from the rest of the sample. The modified FLMP predicted that participant 2 would make fewer mismatch responses than other participants, but that participant 3 would not. The model uses unimodal confusions to predict responses, suggesting that something about participant 2’s responses to unimodal stimuli accounts for the difference in their proportion of mismatch responses. In fact, participant 2 identified visual speech less accurately (23.7%) than any of the other participants (37.2–42.8%). Thus, individual differences in participant 2’s responses to incongruent AV speech appear to result from individual differences in visual-only speech perception.

The modified FLMP did not predict that participant 3 would make fewer mismatch responses than the other participants, suggesting that participant 3’s responses diverged from the rest of the group for reasons unrelated to unimodal speech processing. Participant 3 made auditory responses on nearly all trials with auditory-visual consonant pairs for which the auditory consonant had an alveolar-to-glottal place of articulation and the visual consonant had interdental-to-glottal place of articulation. Participant 3 also made more Other responses than the other participants. Thus, participant 2 was more willing to integrate all middle and back consonants than other subjects.

Large individual differences are a hallmark of AV integration, and some individuals appear to integrate auditory and visual information more optimally (e.g., [37]). Future studies should delve into individual differences in causal inference judgments and their relationship to AV speech perception, more generally.

### Significance

There is a long history of analyzing patterns of consonant confusions to make inferences about speech perception. For example, researchers have used consonant confusions to determine how much information is transmitted by the auditory and visual speech signals, what articulatory features and physical dimensions govern perception of auditory and visual speech, and how the auditory and visual signals contribute to the perception of congruent audiovisual (AV) speech [16, 23, 38]. Many models of AV speech perception were developed and tested using unimodal and congruent AV consonant confusions [15, 25, 26]. Yet, when models incorporate incongruent AV speech, this is typically based on a small set of stimuli varying along a single feature dimension such as voice onset time [8, 10, 12]. The current study is a comprehensive examination of a large set of incongruent AV consonants. The data present an opportunity to test and potentially improve the generalizability of current AV speech integration models.

The results of this study characterize how participants perceive incongruent AV English consonants, including when they judge the consonants as coming from different sources. These data can also guide stimulus selection for studies that aim to use incongruent AV speech stimuli. For example, the authors of this paper have used these data to choose stimuli that consistently resulted in mismatch responses, so that auditory and visual stimuli could vary independently without any concern that participants would fuse the stimuli into an unexpected percept. These large stimulus and data sets (available to anyone who wishes to use them for research purposes) can be used to create more detailed models of AV speech perception and integration.

## Supporting information

S1 Appendix Supplemental vowel analysis. (PDF)

S1 Fig Responses to within- and between-cluster incongruent consonant pairs. Supplemental data corresponding to Fig 4 shows mean and standard deviation (error bars) of the proportion of (A) mismatch responses, (B) auditory responses, (C) visual responses, and (D) Other responses to within- and between- viseme cluster incongruent consonant pairs.
(TIF)

S2 Fig Responses to same-feature and different-feature incongruent AV consonant pairs. Supplemental data corresponding to Fig 7 shows mean and standard deviation (error bars) of the proportion of (A) mismatch responses, (B) auditory responses, (C) visual responses, and (D) Other responses as a function of whether the auditory and visual consonant features (place, voice, manner) were the same or different.
(TIF)

S3 Fig Responses as a function of place of articulation. Supplemental data corresponding to Fig 8 shows mean and standard deviation (error bars) of the proportion of (A) mismatch responses, (B) auditory responses, (C) visual responses, and (D) Other responses as a function of auditory and visual place of articulation.
(TIF)

S4 Fig Vowel-specific responses to unimodal and congruent AV consonants. Responses to (Row 1) auditory-only consonants, (Row 2) congruent AV consonants, (Row 3) the same congruent AV consonants when randomly presented amidst the incongruent stimuli, and (Row 4) visual-only consonants. Columns 1–3 show results for the /ɑ/, /i/, and /u/ contexts, respectively. Responses are averaged across participants.
(TIF)

S5 Fig Vowel-specific proportions of each response type for incongruent syllables. (A) Mean proportion of mismatch, auditory, visual and other responses to incongruent syllables for each vowel context. (B-C) Individual proportions of mismatch, auditory, visual and other responses to incongruent syllables for the /ɑ/, /i/, and /u/ contexts, respectively.
(TIF)

S6 Fig Vowel-specific responses to incongruent AV consonants. Proportion of (Row 1) mismatch responses, (Row 2) auditory responses, (Row 3) visual responses, and (Row 4) Other responses to incongruent AV syllables, as a function of auditory and visual consonant. Columns 1–3 show results for the /ɑ/, /i/, and /u/ contexts, respectively. Responses are averaged across participants.
(TIF)

S7 Fig Vowel-specific responses to within- and between-cluster incongruent consonant pairs. Proportion of each response type to incongruent AV trials for within cluster and between cluster consonant pairs, as a function of vowel context.
(TIF)

S8 Fig Vowel-specific responses as a function of auditory-only accuracy. Proportion of (Row 1) mismatch responses, (Row 2) auditory responses, (Row 3) visual responses, and (Row 4) Other responses as a function of auditory-only identification accuracy. Columns 1–3 show results for the /ɑ/, /i/, and /u/ contexts, respectively.
(TIF)

S9 Fig Vowel-specific responses as a function of visual-only accuracy. Proportion of (Row 1) mismatch responses, (Row 2) auditory responses, (Row 3) visual responses, and (Row 4) Other responses as a function of visual-only identification accuracy. Columns 1–3 show results for the /ɑ/, /i/, and /u/ contexts, respectively.
(TIF)

S10 Fig Vowel-specific responses to same-feature and different-feature incongruent AV consonant pairs. Vowel-specific proportion of each response type as a function of whether auditory and visual consonant features (place, voice, manner) were the same or different.
(TIF)

S11 Fig Vowel-specific responses as a function of place of articulation. Proportion of (Row 1) mismatch responses, (Row 2) auditory responses, (Row 3) visual responses, and (Row 4) Other responses as a function of auditory and visual place of articulation. Columns 1–3 show results for the /ɑ/, /i/, and /u/ contexts, respectively.
(TIF)

S1 Table Paired-samples comparisons between proportions of mismatch responses at each auditory and visual place of articulation for each vowel context. (DOCX)

S2 Table Paired-samples comparisons between proportions of visual responses at each auditory and visual place of articulation for each vowel context. (DOCX)

S3 Table Paired-samples comparisons between proportions of Other responses at each auditory and visual place of articulation in the /i/ context. (DOCX)
