# Supplementary material for: Perception of incongruent audiovisual English consonants
Source: PLoS One. 2019 Mar 21;14(3):e0213588. doi: 10.1371/journal.pone.0213588 (PMC6428273; doi:10.1371/journal.pone.0213588)
Supplement: S1 Appendix — (PDF) [file pone.0213588.s001.pdf]

Perception of auditory-only and congruent AV speech

S4 Fig shows the results for the auditory-only, visual-only, and AV-congruent conditions, combined across subjects, for each vowel context. Results for the /ɑ/, /i/, and /u/ contexts are shown in column 1, 2, and 3, respectively. The mean accuracy in the auditory-only condition (row 1) was 95.41% (S.D. = 3.4%) for /ɑ/, 92.59% (S.D. = 3.15%) for /i/, and 91.68% (S.D. = 3.74%) for /u/. The mean accuracy in the AV condition (row 2) was 97.26% (S.D. = 3.11%) for /ɑ/, 97.5% (S.D. = 2.95%) for /i/, and 97.5% (S.D. = 3.57%) for /u/.

Congruent AV syllables were also presented mixed with the incongruent AV syllables (row 3). Participants made 5.52%, 4.02%, and 4.89% more errors on congruent trials randomly presented amidst the incongruent stimulus trials than when only congruent syllables were presented, in the /ɑ/, /i/, and /u/ vowel contexts respectively. The additional errors were reports that 4.24%, 3.59%, and 3.92% of congruent syllables did not match when presented amidst the incongruent stimulus trials, in the /ɑ/, /i/, and /u/ vowel contexts, respectively. There was no significant difference across vowel contexts in the proportion of congruent trials on which participants made mismatch responses.

Perception of visual-only speech

In the visual-only condition (S4 Fig, row 3), participants correctly identified 40.34% (S.D. = 5.42%) of consonants in the /ɑ/ context, 39.61% (S.D. = 6.86%) of consonants in the /i/ context, and 34.94% (S.D. = 8.21%) of consonants in the /u/ context. A one-way repeated measures ANOVA indicated a significant difference in visual-only consonant identification accuracy across vowel contexts, $F = 6.117$, $p = 0.0123$, df = 2, 24. Paired-samples t-tests with Bonferroni corrections for multiple comparisons (critical p-value = 0.0167) indicated significantly poorer accuracy in the /u/ context than in the /i/ context, $t = 3.137$, $p = 0.0164$, df = 7, and marginally poorer accuracy in the /u/ context than in the /ɑ/ context, $t = 2.651$, $p = 0.0329$, df = 7.

Perception of incongruent AV speech

S5 Fig (a) shows the mean proportion of each type of response, in each of the vowel contexts. Individual data for each vowel context are shown in S5 Fig (b-d). Differences in these proportions across vowel contexts are discussed for each response type below.

Analysis of responses to incongruent AV speech

S6 Fig shows the proportion of mismatch responses (row 1), auditory responses (row 2), visual responses (row 3) and other responses (row 4) for each auditory-visual syllable pair, in each vowel context. Results for the /ɑ/, /i/, and /u/ contexts are shown in columns 1, 2, and 3, respectively. In the main text, we reported the results of a series of analyses to determine what features predicted how participants perceive incongruent consonants. In this supplement, we examined differences in these predictions across vowel context.

Viseme clusters

S7 Fig shows the proportion of mismatch, auditory, visual, and Other responses for auditory and visual consonants in the same viseme cluster (Table 1) as compared to auditory and visual consonants from different viseme clusters, for each of the three vowel contexts. We used two-way repeated measures ANOVA to examine whether the effect of viseme cluster depended on vowel context. We followed-up significant interactions with a series of paired-samples t-tests for each vowel-context, comparing the proportion of responses for within- and between-viseme-cluster consonant pairs. Bonferroni corrections for multiple comparisons were applied (critical p = 0.0167). Results are discussed for each response type below.

Unimodal accuracy

S8 and S9 Figs show the relationship between the proportion of each response type and auditory-only and visual-only accuracy, respectively, for each vowel context. Each row represents a response type (mismatch, auditory, visual, and Other) and each column represents a vowel context (/ɑ/, /i/, and /u/). In the main text, we examined the relationship between unimodal accuracy and responses to incongruent AV stimuli, with both measures averaged across vowel context. Specifically, we used Kendall's tau to examine the relationship between the proportion of mismatch, auditory, visual, and Other responses for consonants with auditory-only and visual-only accuracy. Here, we use the same test for each vowel context, to determine whether the effects of unimodal auditory and unimodal visual accuracy are consistent across vowel contexts.

Congruency of speech features

In the main text, we examined whether responses differed based on the congruency of place, voice, and manner features. Feature assignment for each consonant is shown in Table 2. Here, we used two-way repeated measures ANOVA with effects of vowel context and speech feature congruency to determine whether the effect of place, manner, and voice congruency differed across vowel contexts. We followed-up significant interactions with a series of paired-samples t-tests for each vowel-context, comparing the proportion of responses for same-feature and different-feature pairs. Bonferroni corrections for multiple comparisons were applied (critical p = 0.0167). Results are show in S10 Fig and discussed for each response type below.

Place of articulation

S11 Fig shows the proportion of mismatch, auditory, visual, and Other responses as a function of auditory and visual place of articulation. Each row represents a response type (mismatch, auditory, visual, and Other) and each column represents a vowel context (/ɑ/, /i/, and /u/). Place assignment for each consonant is shown in Table 2. We used three-way repeated measures ANOVAs to determine whether the effects of auditory and visual consonant place and interaction of auditory and visual consonant place depended on vowel context. When auditory and/or visual place interacted with vowel context, we completed two-way repeated measures ANOVAs with effects of auditory and visual place for each vowel context. One-way ANOVAs were used to explore significant interactions between auditory and visual place, and paired-samples t-tests with Bonferroni corrections for multiple comparisons were used to explore significant effects (critical $p = 0.0167$). Results are discussed for each response type below.

Mismatch responses

In all three vowel conditions, participants noticed the mismatch between auditory and visual signals for the majority of incongruent AV consonant pairs (64%, 58%, and 55% in the /ɑ/, /i/, and /u/ contexts, respectively) (S5 Fig). One-way repeated-measures ANOVA indicated a significant effect of vowel context on the proportion of mismatch responses, $F = 6.242$, $p = 0.0115$, df = 2,21. Paired-samples t-tests with Bonferroni corrections for multiple comparisons (critical p-value = 0.0167) indicated a significantly higher proportion of mismatch responses in the /ɑ/ context than in the /u/ context, $t = 3.591$, $p = 0.0088$, df = 7.

Viseme clusters

In the main text, we reported that participants were more likely to notice the mismatch when the auditory and visual consonants belonged to different viseme clusters. Two-way repeated measures ANOVA confirmed a significant effect of within- vs. between-viseme-cluster pairs, $F = 223.362$, $p < 0.0001$, df. = 1, 35. This effect did not interact with vowel context (S7 Fig).

Unimodal Accuracy

In the main text, we reported that participants were more likely to notice the mismatch between consonants if the visual consonant was identified with high accuracy. This effect was consistent across all three vowel contexts: for /ɑ/, $r_\tau = 0.2515$, $p < 0.0001$, for /i/, $r_\tau = 0.2541$, $p < 0.0001$, and for /u/, $r_\tau = 0.2228$, $p < 0.0001$ (S9). Although not significant overall, results in the /i/ context indicated that participants were more likely to notice the mismatch between consonants if the auditory consonant was identified with high accuracy, $r_\tau = 0.165$, $p < 0.0001$.

Congruency of speech features

As reported in the main text, participants were more likely to notice the mismatch when the auditory and visual consonants differed in place and manner of articulation, and when the auditory and visual consonants were the same on the voicing feature. Two-way repeated measures ANOVA indicated significant effects of vowel context, $F \geq 9.306$, $p = 0.0006$, df = 2, and feature congruency, $F \geq 10.058$, $p = 0.0032$, df = 2, 35 for all three features. For all features, there were no interactions of vowel context and feature congruency, suggesting that the effects of place, manner, and voice congruency on mismatch responses were consistent across vowel contexts (S10 Fig).

Place of articulation

The first row of S11 Fig shows the effects of auditory and visual place of articulation on mismatch responses, as a function of vowel context. Patterns appear similar across vowel contexts and are consistent with the results reported in the main text. There are greater proportions of mismatch responses for consonants produced further apart and greater proportions of mismatch responses for middle consonants paired with front consonants than middle consonants paired with back consonants. Statistical analysis revealed a small but significant three-way interaction of vowel context, auditory place, and visual place on mismatch responses, $F = 2.350$, $p = 0.0199$, df = 8, 182. For all vowel contexts, there were significant interactions of auditory and visual place of articulation, $F \geq 20.542$, $p < 0.0001$, df = 4, 56. Additionally, all of the one-way ANOVA's comparing auditory place at each visual place and comparing visual place at each auditory place were significant, $F \geq 14.76$, $p \leq 0.0003$. The mean data showed the same

patterns across all three vowel contexts, but the significance of each paired-samples contrast varied across vowel contexts. The results of these t-tests can be found in S1 Table.

Summary

To summarize, the relation between the predictive variables and the proportion of mismatch responses was generally consistent across vowel contexts and with the findings reported in the main text. By analyzing the relationships as a function of vowel context, we found one additional significant result. Only in the /i/ context, mismatch responses were more common for higher accuracy auditory consonants. However, this may just reflect the slightly increased variability of visual-only accuracy in the /i/ context.

Auditory responses

When participants perceived the auditory and visual information as coming from a single source, they were most likely to report the auditory syllable (30%, 32%, and 38% in the /ɑ/, /i/, and /u/ contexts, respectively). One-way repeated measures ANOVA indicated a significant effect of vowel context on the proportion of auditory responses, $F = 8.721$, $p = 0.0035$, df = 2,21. Auditory responses were more common for the /u/ context than the /ɑ/ context, $t = -3.8441$, $p = 0.0063$, df = 7, and /i/ context, $t = -3.6313$, $p = 0.0084$, df = 7.

Viseme clusters

In the main text, we reported that auditory responses were more common when the auditory and visual consonants belonged to the same viseme cluster. Two-way repeated measures ANOVA confirmed a significant effect of the within- vs. between-viseme-cluster comparison, $F = 418.45$, $p < 0.0001$, df. = 1, 35. This effect did not interact with vowel context (S7 Fig).

Unimodal Accuracy

In the main text, we reported that auditory responses were higher for auditory consonants with high auditory-only accuracy. Conversely, auditory responses were higher for visual consonants with low visual-only accuracy. The effect of auditory consonant accuracy was consistent across vowel contexts: for /ɑ/, $r_\tau = 0.0808$, $p = 0.0213$, for /i/, $r_\tau = 0.1986$, $p < 0.0001$, and for /u/, $r_\tau = 0.3020$, $p < 0.0001$ (S8 Fig). The effect of visual consonant accuracy was also consistent across vowel contexts: for /ɑ/, $r_\tau = -0.2323$, $p < 0.0001$, for /i/, $r_\tau = -0.1651$, $p < 0.0001$, and for /u/, $r_\tau = -0.1774$, $p < 0.0001$ (S9 Fig).

Congruency of speech features

As reported in the main text, auditory responses were more common when the auditory and visual had the same place and manner of articulation, and when the auditory and visual consonants differed on the voicing feature. Across all three features, the two-way repeated-measures ANOVA indicated main effects of vowel context, $F \geq 9.189$, $p \leq 0.0006$, df = 2, 35 and feature congruency, $F \geq 20.126$, $p < 0.0001$, df = 1, 35. For all features, there was no interaction of vowel context and feature congruency, suggesting that the effects of place, manner, and voice congruency on mismatch responses were consistent across vowel contexts (S10 Fig).

Place of articulation

The second row of S11 Fig shows the effects of auditory and visual place of articulation on auditory responses, as a function of vowel context. Patterns appear similar across vowel contexts. Three-way ANOVA confirmed the significant interaction of auditory and visual place, $F = 93.574$, $p < 0.0001$. Vowel context did not interact with auditory and/or visual place, confirming that the effects reported in the main text were consistent across vowel contexts.

Summary

In summary, the relation between the predictive variables and the proportion of auditory responses was completely consistent across vowel contexts and with the findings reported in the main text.

Visual responses

Participants only reported the visual syllable 1.0%, 1.4%, and 0.9% of the time in the /ɑ/, /i/, and /u/ contexts, respectively. One-way repeated measures ANOVAs indicated a significant effect of vowel context, $F = 23.23$, $p <0.0001$, df = 2,21. Visual responses were significantly more common in the /i/ context than the /ɑ/ context, $t = -4.5225$, $p = 0.0027$, df = 7, and /u/ context, $t = 7.4584$, $p = 0.0001$, df = 7.

Viseme clusters

In the main text, we reported that there was no significant difference between the proportion of visual responses to within-viseme-cluster and between-viseme-cluster pairs. The two-way ANOVA also failed to find a significant overall effect of within- vs. between-viseme-cluster pairs. However, there was a significant interaction of viseme cluster and vowel context, $F = 11.755$, $p = 0.0001$, df = 2,35. Paired samples t-tests indicated that visual responses were more common for between-viseme-cluster pairs than within-viseme cluster pairs in the /i/ context, $t = -3.566$, $p = 0.0091$, df = 7, and the /u/ context, $t = -3.352$, $p = 0.0122$, df = 7 (S7 Fig).

Unimodal Accuracy

In the main text, we reported a weak but significant relationship between visual responses and auditory and visual consonant accuracy. Visual responses were more common for auditory consonants with lower auditory-only identification accuracy and for visual consonants with lower visual-only identification accuracy. The effect of auditory consonant accuracy was consistent across all vowel contexts: for /ɑ/, $r_\tau = -0.09$, $p < 0.0302$, for /i/, $r_\tau = -0.1073$, $p = 0.009$, and for /u/, $r_\tau = -0.2000$, $p < 0.0001$ (S8 Fig). However, the effect of visual accuracy was significant only in the /i/ context, $r_\tau = 0.0962$, $p = 0.0009$ (S9 Fig).

Congruency of speech features

In the main text, we reported that visual responses were more common for incongruent consonants pairs with the same manner and incongruent consonant pairs with same voicing. We used two-way repeated measures ANOVA with effects of vowel context and speech feature congruency to determine whether the effect of place, manner, and voice congruency on visual responses differed across vowel contexts.

For manner of articulation, there were significant effects of vowel context, F = 10.85, p = 0.0002, df = 2,35, and manner congruency, F = 204.37, p < 0.0001, df = 1,35. There was also a significant interaction of vowel context and manner congruency, F = 16.71, p < 0.0001, df = 2,35. Paired samples t-tests indicated that the effect of manner was significant in all three vowel contexts: the /ɑ/ context, t = 4.444, p = 0.003, df = 7, the /i/ context, t = 11.683, p < 0.0001, df = 7, and the /u/ context, t = 4.597, p = 0.0025. Across all three vowel contexts, visual responses were more common when manner was the same across the auditory and visual consonants. However, these effects appear to be stronger in the /i/ context.

For voice, there were significant effects of vowel context, F = 3.814, p = 0.0317, df = 2,35, and voice congruency, F = 112.529, p < 0.0001, df = 1,35. There was also a significant interaction of vowel context and voice congruency, F = 4.644, p = 0.0163, df = 2,35. Across all vowel contexts, there was a trend to observe more visual responses when voicing was the same for the auditory and visual consonants. This effect was strongest in the /i/ context, t = 9.184, p < 0.0001, was significant in the /u/ context, t = 4.514, p = 0.0028, and just failed to reach significance following Bonferroni corrections in the /ɑ/ context, t = 3.103, p = 0.0172. It's possible that the voice and manner congruency effects were greater for the /i/ context simply because visual responses were more common in the /i/ context.

For place of articulation, there was no main effect of congruency. However, there was a significant interaction of vowel context and place congruency, F = 17.773, p < 0.0001, df = 2, 35. Paired samples t-tests indicated that the effect of place was only significant in the /ɑ/ context, t = 3.555, p = 0.0093, df = 7. In the /ɑ/ context, visual responses were more common for auditory and visual consonants with the same place of articulation. Recall that we used a relatively broad front-middle-back distinction for place of articulation. Table 3 shows that the most common visual responses were for consonants differing only slightly in place of articulation. The place effect on visual responses may have been significant only in the /ɑ/ context because visual consonants were easier to identify in the /ɑ/ context.

Place of articulation

The third row of S11 Fig shows the effects of auditory and visual place of articulation on visual responses, as a function of vowel context. Statistical analysis revealed a three-way interaction of vowel context, auditory place, and visual place on visual responses, F = 19.314, p < 0.0001, df = 8, 182. For all vowel contexts, there were significant interactions of auditory and visual place of articulation, F ≥ 6.405, p ≤ 0.0002, df = 4, 56. The one-way ANOVA's comparing auditory place at each visual place were significant for all vowel contexts for front visual place, F ≥ 10.65, p ≤ 0.0015, df = 2,14 and middle visual place, F ≥ 5.281, p ≤ 0.0195, df = 2,14. In the /ɑ/ context, the effect of auditory place was also significant for back visual place, F = 5.092, p = 0.0218, df = 2,14. Likewise, for all vowel contexts, the one-way ANOVA's comparing visual place at each auditory place were significant for all vowel contexts for front auditory place, F ≥ 13.84, p < 0.0005, df = 2,14 and auditory middle place, F ≥ 6.657, p ≤ 0.009, df = 2,14. In the /i/ context, the effect of visual place was also significant for back auditory place, F = 4.724, p = 0.027, df = 2,14.

The results of follow-up, paired-samples t-tests can be found in S2 Table. In general, across all vowel contexts, visual responses were highly uncommon with either back auditory and back visual vowels. Vowel-related differences in the effects of visual place were observed for front and middle auditory and visual consonant pairs. Specifically, S11 Fig shows that in the /ɑ/ context, auditory front/visual middle consonant pairs resulted in more visual responses than auditory front/visual front consonant pairs. The reverse was true in the /i/ context, and on difference was observed in the /u/ context. Some additional differences between middle/front and front/middle

combinations—compared to front/front and middle/middle combinations—were observed only in the /i/ context.

Summary

In summary, the relation between the predictive variables and the proportion of visual responses varied somewhat across viseme categories. The relationship between visual responses and viseme categories was only significant for the /i/ and /u/ vowel contexts, the relationship between visual responses and accuracy of unimodal identification of the visual consonant was only significant for the /i/ context, and the strength of the voice and manner congruency effect were greater in the /i/ context. An effect of place congruency that was not present in the overall data emerged for the /ɑ/ context. Finally, some differences in the effects of auditory and visual place of articulation were only significant for the /i/ context. This variation in findings across vowel contexts might reflect the small proportion of visual responses throughout the experiment.

Other responses

The remaining 4.4%, 8.4% and 5.5% of responses in the /ɑ/, /i/, and /u/ contexts, respectively, fell into the “Other” category. Other responses include those that we traditionally think of as “integration” of incongruent AV speech signals: McGurk-like fused and combined percepts. One-way repeated measures ANOVA indicated a significant effect of vowel context on the proportion of other responses, $F = 10.27$, $p = 0.0018$, $df = 2,21$. Other responses were significantly more common in the /i/ context than the /ɑ/ context, $t = -3.5186$, $p = 0.0097$, $df = 7$, and /u/ context, $t = 3.5551$, $p = 0.0093$, $df = 7$.

Viseme clusters

In the main text, we reported that Other responses were significantly more common for auditory and visual consonants from different viseme clusters. Two-way repeated measures ANOVA confirmed this effect, $F = 20.381$, $p < 0.0001$, $df = 1,35$. This effect did not interact with vowel context, suggesting that it was consistent across vowel contexts (S7 Fig).

Unimodal Accuracy

In the main text, we reported that Other responses were more common for auditory consonants with lower levels of auditory-only identification accuracy and did not depend on the visual-only identification accuracy of the visual consonant. The effect of auditory accuracy was consistent across all three vowel contexts: for /ɑ/, $r_\tau = -0.3948$, $p < 0.0001$, for /i/, $r_\tau = 0\text{-}.4808$, $p < 0.0001$, and for /u/, $r_\tau = -0.6521$, $p < 0.0001$. No effect of visual accuracy was observed for any vowel context.

Congruency of speech features

In the main text, we reported that Other responses were more common for incongruent consonant pairs with the same manner and incongruent consonant differing in voicing. Two-way repeated measures ANOVA confirmed these results, with significant effects of vowel context, $F \geq 9.208$, $p \leq 0.0006$, $df = 2,35$, manner congruency, $F = 13.95$, $p = 0.0007$, $df = 1,35$, and voice congruency, $F = 19.787$, $p < 0.0001$, $df = 1,35$. In both cases, there were no significant interactions

of vowel context and feature congruency, suggesting that the effects of manner and voice congruency on Other responses were consistent across vowel contexts (S10 Fig).

Two-way ANOVA also revealed a significant effect of place congruency, $F = 7.583$, $p = 0.0093$, $df = 1,35$, and a significant interaction of vowel context and place congruency, $F = 3.492$, $p <= 0.0414$, $df = 2,35$. To explore this interaction, we completed paired samples t-tests comparing the proportion of visual responses for same-place and different-place pairs in each vowel context. None of the comparisons were significant after Bonferroni corrections for multiple comparisons (critical $p = 0.0167$), but there was a non-significant trend for more other responses to different-place pairs in the /i/ context, $t = -2.393$, $p = 0.0476$. The effect of congruency was likely stronger for the /i/ vowel context because there was also a higher proportion of Other responses.

Place of articulation

The last row of S11 Fig shows the effects of auditory and visual place of articulation on visual responses, as a function of vowel context. Statistical analysis revealed a three-way interaction of vowel context, auditory place, and visual place on visual responses, $F = 3.199$, $p = 0.0020$, $df = 8, 182$. To explore the interaction, we completed a two-way ANOVA for each vowel context. No significant effects or interactions were observed in the /ɑ/ context. In the /i/ context, there was a significant effect of auditory place, $F = 18.906$, $p < 0.0001$, $df = 2,56$, and a significant interaction of auditory and visual place, $F = 7.821$, $p < 0.0001$, $df = 4,56$. Follow-up t-tests can be found in S3 Table. To summarize, in the /i/ context, Other responses were most common for auditory front/visual middle consonant pairs. They were also more common for auditory front/visual back and auditory middle/visual front consonant pairs than some other consonant pairs. In the /u/ context, there was only a significant effect of auditory place, $F = 13.607$, $p < 0.0001$, $df = 2,56$. Although the mean data suggest more Other responses for more front places of articulation, none of the paired comparisons between auditory places were significant after Bonferroni correction for multiple comparisons.

Summary

In summary, the relation between the predictive variables and the proportion of Other responses was generally consistent across vowel contexts and with the findings reported in the main text. The exception was in the place of articulation analysis, which indicated effects of auditory place in the /i/ context, an interaction of auditory and visual place in the /i/ context, and no effect of place in the /ɑ/ context. These differences across vowel contexts may reflect the small proportion and highly variability of Other responses.

General Summary

We expected to observe some differences in consonant perception as a function of vowel context, because many visual consonants are easier to discriminate with the open vowel /ɑ/ than the close, back vowel /u/ [13]. Vowel context could thus effect the salience and redundancy of information in the visual modality and the redundancy of information across modalities. In general, visual-only accuracy and the proportion of each response type varied across vowel contexts. However, the relationship between predictive variables and proportions of mismatch, auditory and Other responses was typically the same across vowels. Many vowel-related differences were found for visual responses, likely due to their relatively small representation of visual responses in the data. The stability of the relationship between the predictive variables and both causal inference judgments and incongruent AV speech responses supports the generalizability of these relationships.
