# Supplementary figures and images for: Perception of incongruent audiovisual English consonants
Source: PLoS One. 2019 Mar 21;14(3):e0213588. doi: 10.1371/journal.pone.0213588 (PMC6428273; doi:10.1371/journal.pone.0213588)

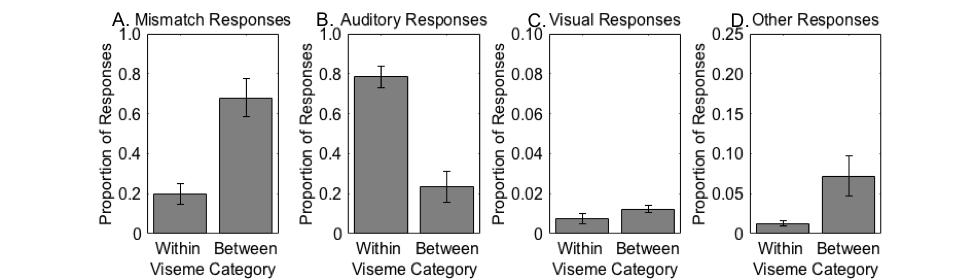

Supplement: S1 Fig — Supplemental data corresponding to Fig 4 shows mean and standard deviation (error bars) of the proportion of (A) mismatch responses, (B) auditory responses, (C) visual responses, and (D) Other responses to within- and between- viseme cluster incongruent consonant pairs. (TIF) [file pone.0213588.s002.tif]

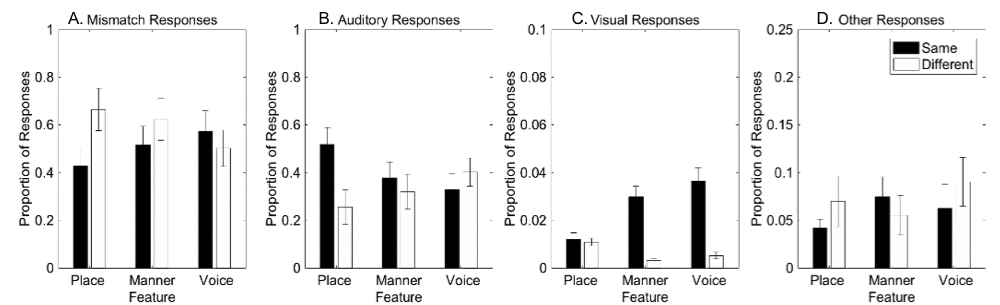

Supplement: S2 Fig — Supplemental data corresponding to Fig 7 shows mean and standard deviation (error bars) of the proportion of (A) mismatch responses, (B) auditory responses, (C) visual responses, and (D) Other responses as a function of whether the auditory and visual consonant features (place, voice, manner) were the same or different. (TIF) [file pone.0213588.s003.tif]

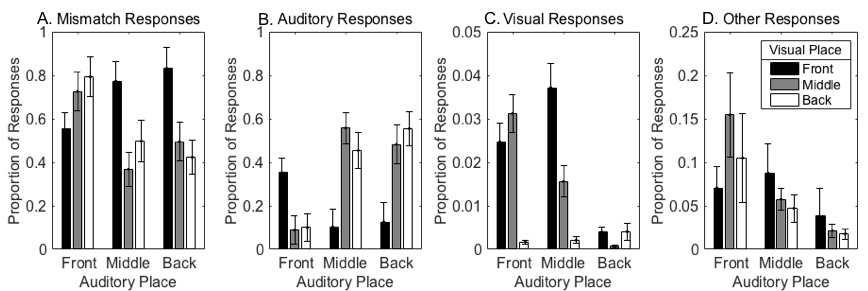

Supplement: S3 Fig — Supplemental data corresponding to Fig 8 shows mean and standard deviation (error bars) of the proportion of (A) mismatch responses, (B) auditory responses, (C) visual responses, and (D) Other responses as a function of auditory and visual place of articulation. (TIF) [file pone.0213588.s004.tif]

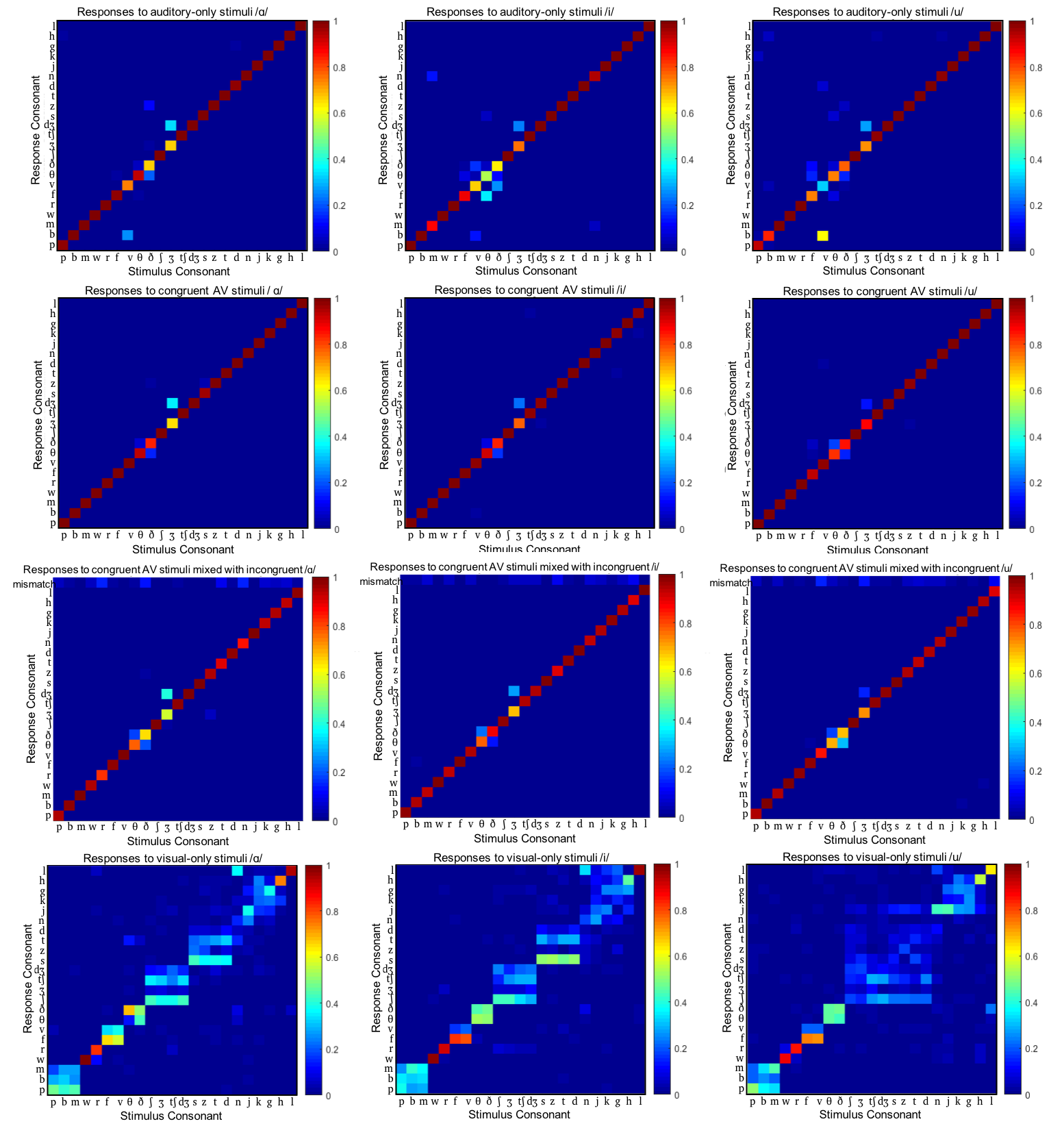

Supplement: S4 Fig — Responses to (Row 1) auditory-only consonants, (Row 2) congruent AV consonants, (Row 3) the same congruent AV consonants when randomly presented amidst the incongruent stimuli, and (Row 4) visual-only consonants. Columns 1–3 show results for the /ɑ/, /i/, and /u/ contexts, respectively. Responses are averaged across participants. (TIF) [file pone.0213588.s005.tif]

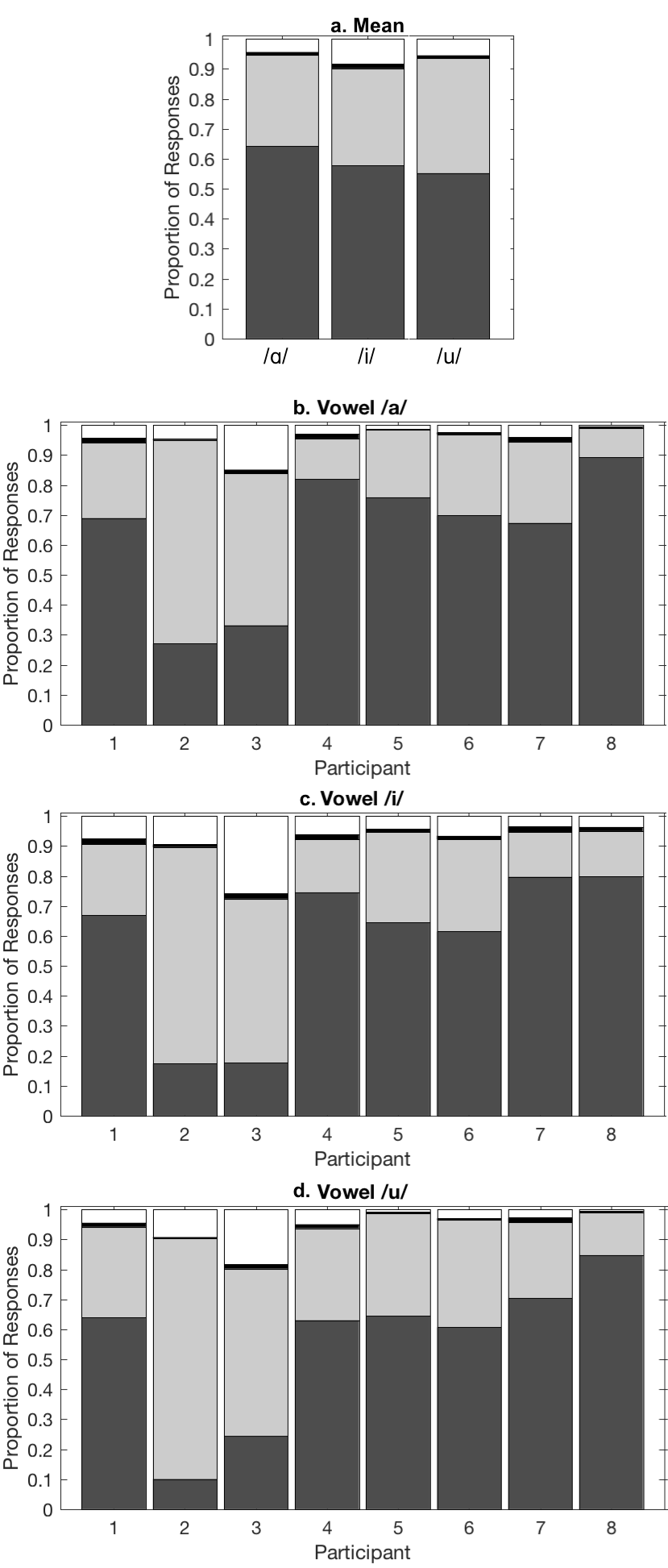

Supplement: S5 Fig — (A) Mean proportion of mismatch, auditory, visual and other responses to incongruent syllables for each vowel context. (B-C) Individual proportions of mismatch, auditory, visual and other responses to incongruent syllables for the /ɑ/, /i/, and /u/ contexts, respectively. (TIF) [file pone.0213588.s006.tif]

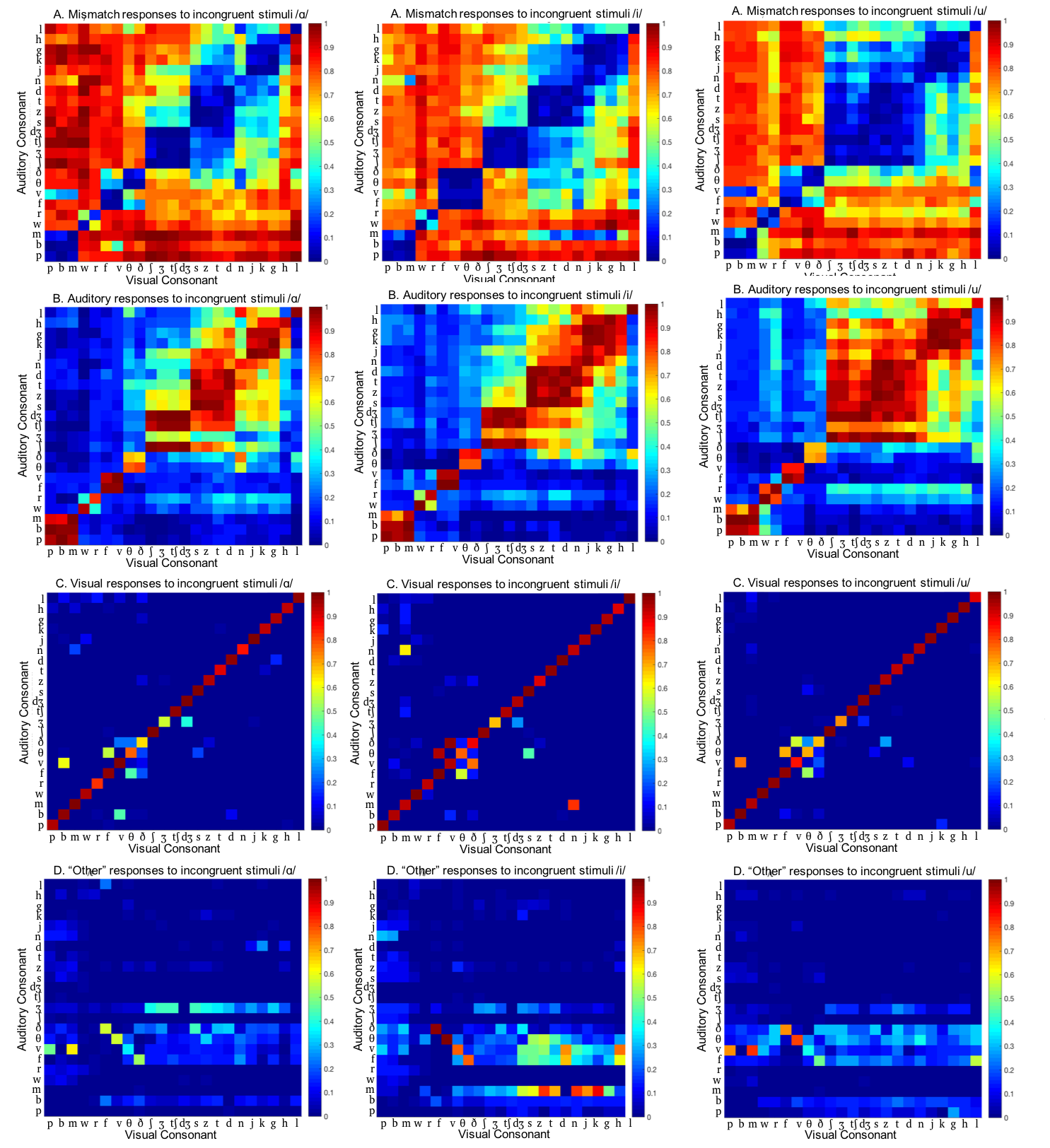

Supplement: S6 Fig — Proportion of (Row 1) mismatch responses, (Row 2) auditory responses, (Row 3) visual responses, and (Row 4) Other responses to incongruent AV syllables, as a function of auditory and visual consonant. Columns 1–3 show results for the /ɑ/, /i/, and /u/ contexts, respectively. Responses are averaged across participants. (TIF) [file pone.0213588.s007.tif]

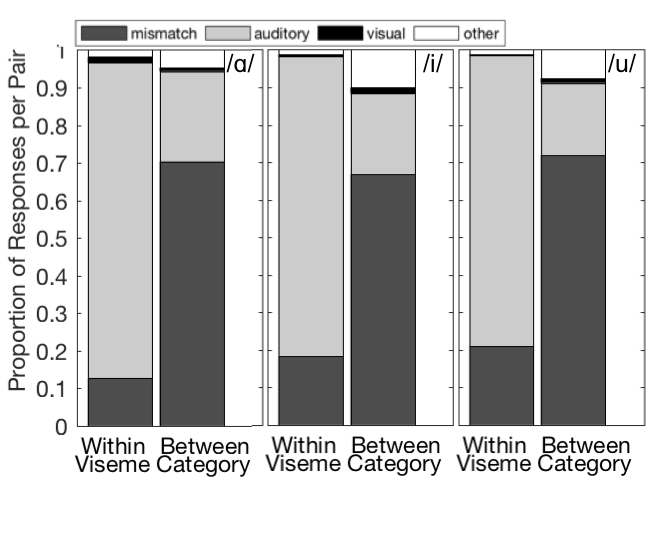

Supplement: S7 Fig — Proportion of each response type to incongruent AV trials for within cluster and between cluster consonant pairs, as a function of vowel context. (TIF) [file pone.0213588.s008.tif]

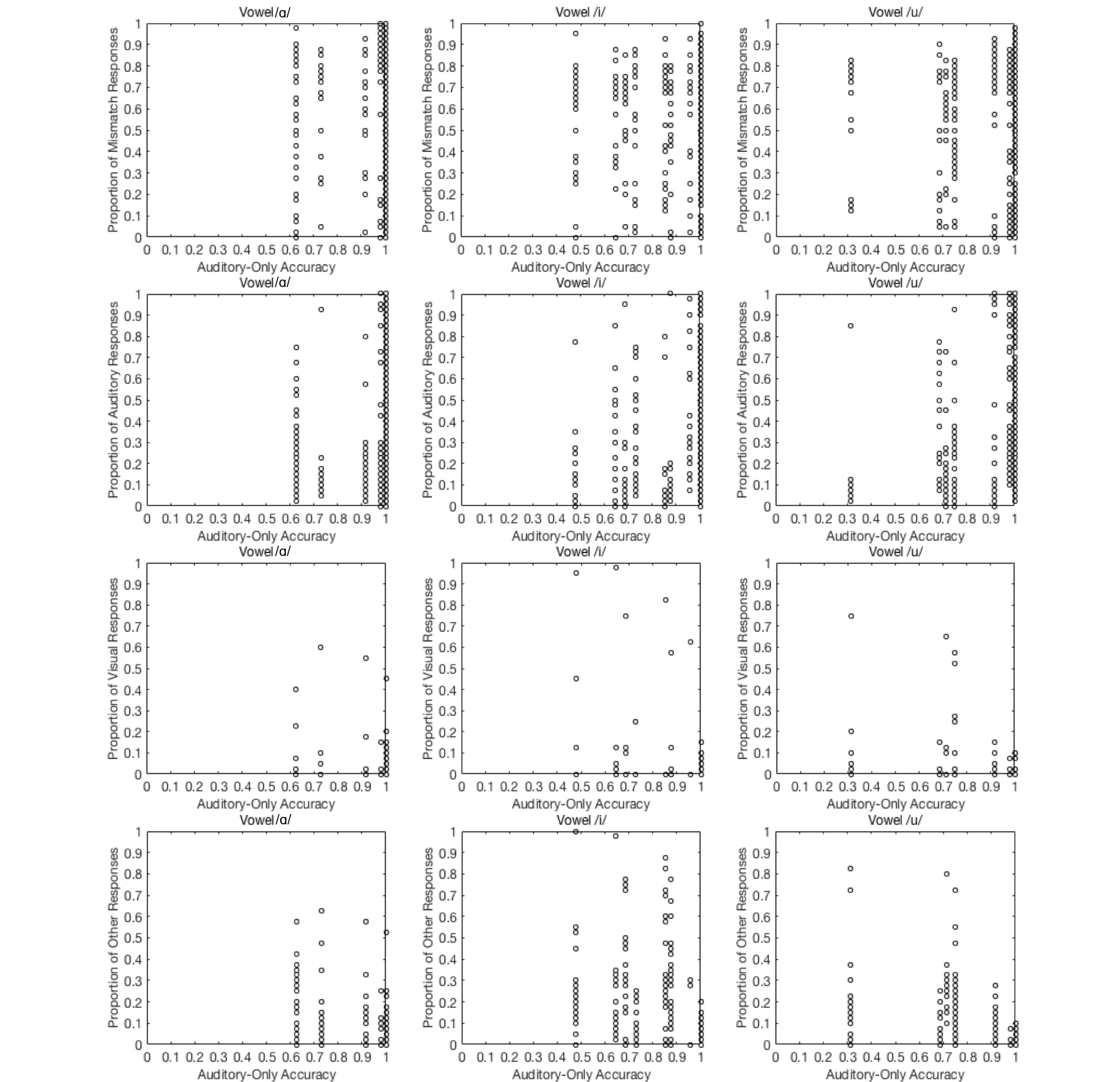

Supplement: S8 Fig — Proportion of (Row 1) mismatch responses, (Row 2) auditory responses, (Row 3) visual responses, and (Row 4) Other responses as a function of auditory-only identification accuracy. Columns 1–3 show results for the /ɑ/, /i/, and /u/ contexts, respectively. (TIF) [file pone.0213588.s009.tif]

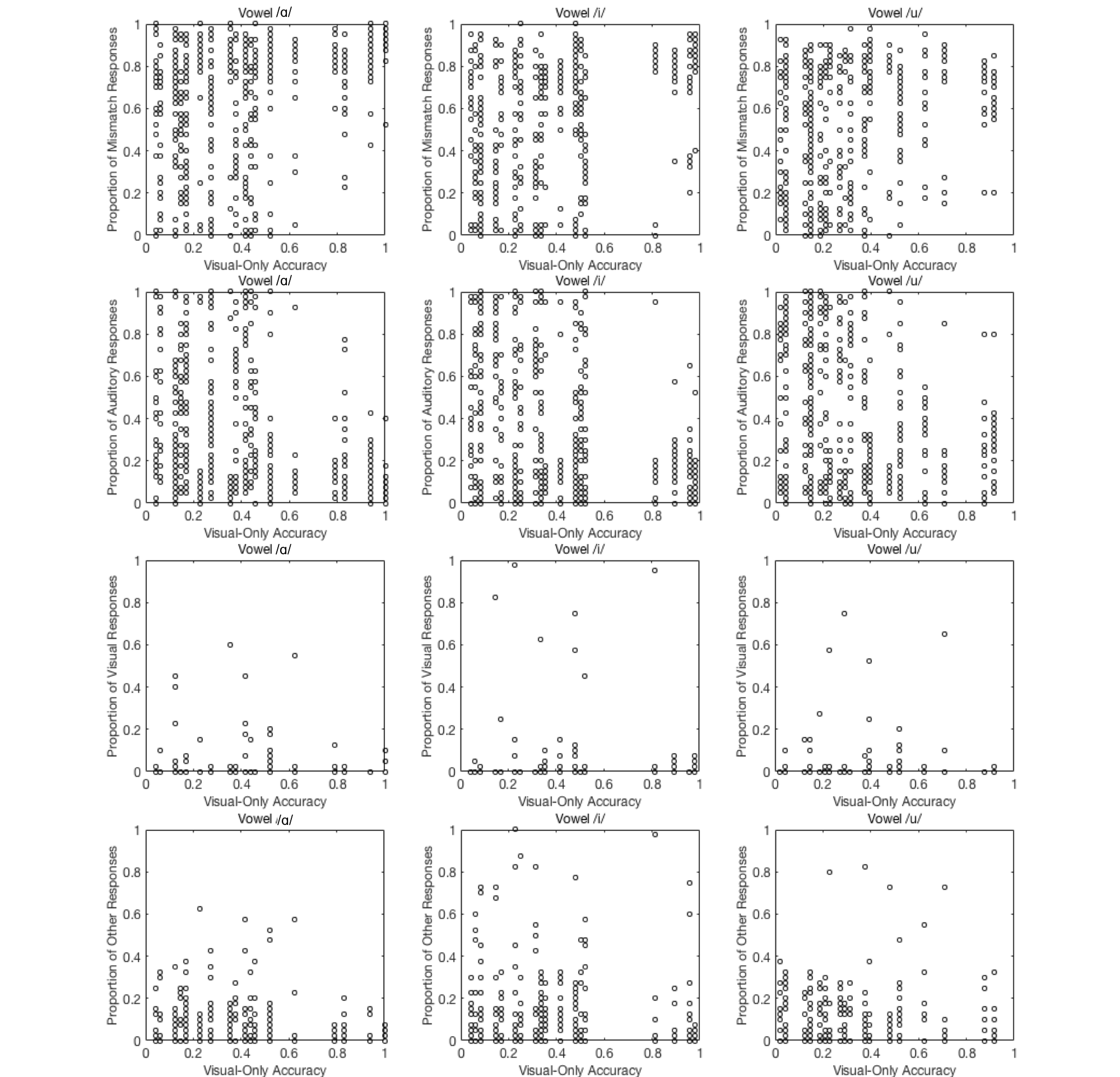

Supplement: S9 Fig — Proportion of (Row 1) mismatch responses, (Row 2) auditory responses, (Row 3) visual responses, and (Row 4) Other responses as a function of visual-only identification accuracy. Columns 1–3 show results for the /ɑ/, /i/, and /u/ contexts, respectively. (TIF) [file pone.0213588.s010.tif]

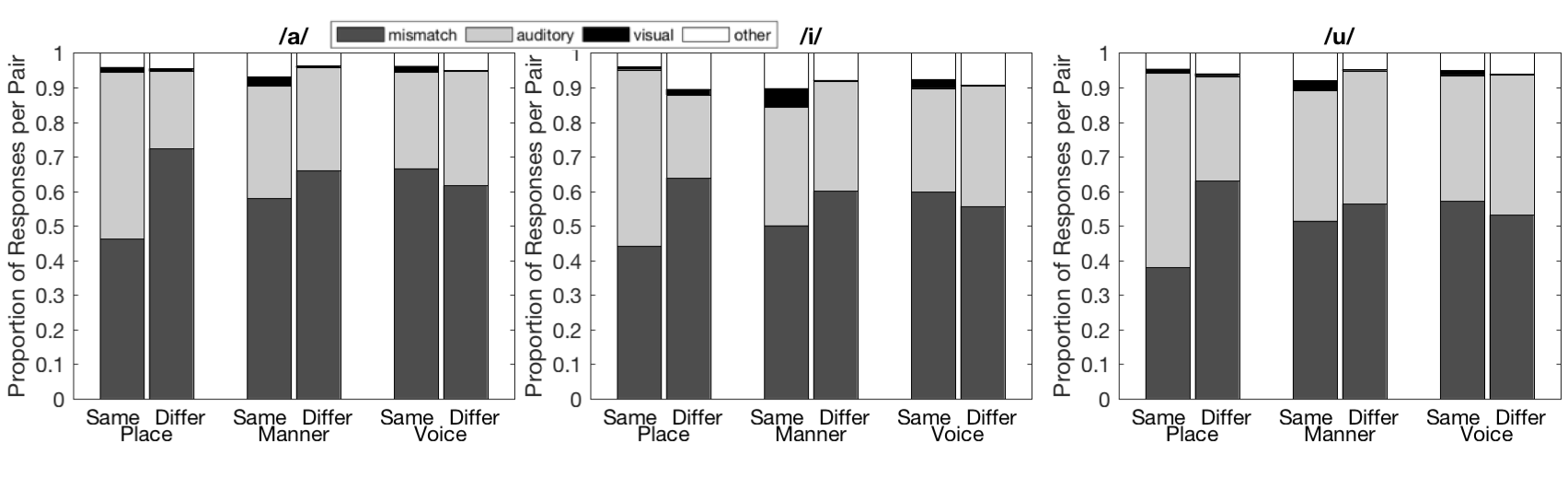

Supplement: S10 Fig — Vowel-specific proportion of each response type as a function of whether auditory and visual consonant features (place, voice, manner) were the same or different. (TIF) [file pone.0213588.s011.tif]

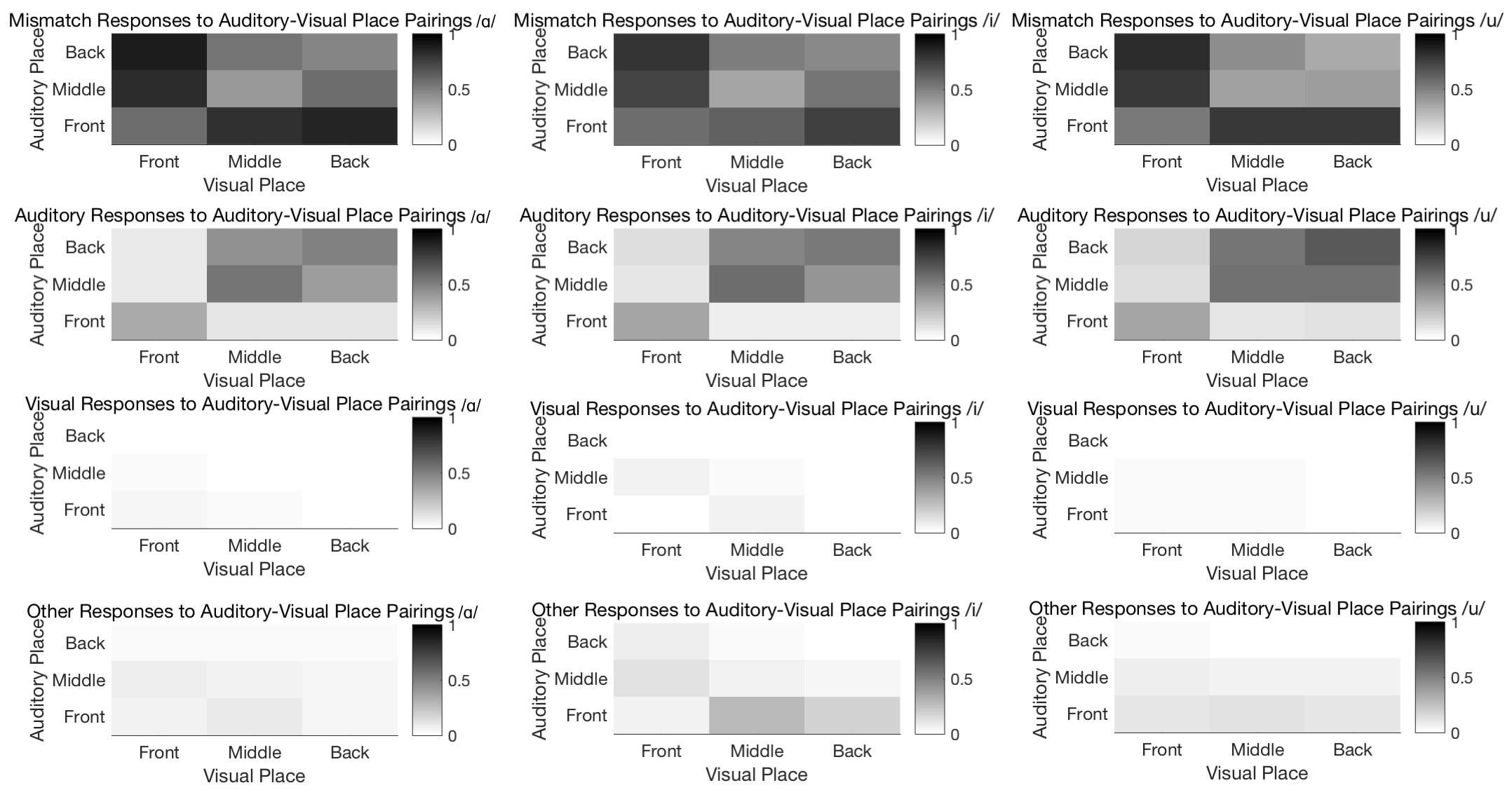

Supplement: S11 Fig — Proportion of (Row 1) mismatch responses, (Row 2) auditory responses, (Row 3) visual responses, and (Row 4) Other responses as a function of auditory and visual place of articulation. Columns 1–3 show results for the /ɑ/, /i/, and /u/ contexts, respectively. (TIF) [file pone.0213588.s012.tif]
