# Supplementary material for: Perception of incongruent audiovisual English consonants
Source: PLoS One. 2019 Mar 21;14(3):e0213588. doi: 10.1371/journal.pone.0213588 (PMC6428273; doi:10.1371/journal.pone.0213588)
Supplement: S1 Table — (DOCX) [file pone.0213588.s013.docx]

**S1 Table. Results of paired-samples t-tests comparing the proportion of mismatch responses across different auditory and visual places of articulation.**

| Consonant 1 | Consonant 2 | /ɑ/ t-stat, p-value | | /i/ t-stat, p-value | | /u/ t-stat, p-value | |
| --- | --- | --- | --- | --- | --- | --- | --- |
| front-front | front-mid | -5.539 | 0.0009 | -1.103 | 0.3066 | -8.059 | 0.0001 |
| front-front | front-back | -9.700 | < 0.0001 | -4.199 | 0.0040 | -6.414 | 0.0004 |
| front-mid | front-back | -3.111 | 0.0171 | -6.972 | 0.0002 | -0.686 | 0.5147 |
| mid-front | mid-mid | 10.195 | < 0.0001 | 6.948 | 0.0002 | 6.144 | 0.0005 |
| mid-front | mid-back | 4.328 | 0.0034 | 2.754 | 0.0283 | 5.102 | 0.0014 |
| mid-mid | mid-back | -6.098 | 0.0005 | -4.808 | 0.0019 | -1.071 | 0.3196 |
| back-front | back-mid | 5.890 | 0.0006 | 3.812 | 0.0066 | 5.668 | 0.0008 |
| back-front | back-back | 8.255 | 0.0001 | 4.950 | 0.0017 | 6.558 | 0.0003 |
| back-mid | back-back | 3.376 | 0.0118 | 1.390 | 0.2072 | 6.613 | 0.0003 |
| front-front | mid-front | -14.728 | < 0.0001 | -8.723 | 0.0001 | -6.232 | 0.0004 |
| front-front | back-front | -17.541 | < 0.0001 | -6.314 | 0.0004 | -6.008 | 0.0005 |
| mid-front | back-front | -4.840 | 0.0019 | -2.910 | 0.0226 | -3.334 | 0.0125 |
| front-mid | mid-mid | 9.727 | < 0.0001 | 15.683 | < 0.0001 | 8.312 | 0.0001 |
| front-mid | back-mid | 5.394 | 0.0010 | 3.212 | 0.0148 | 7.836 | 0.0001 |
| mid-mid | back-mid | -8.936 | < 0.0001 | -5.860 | 0.0006 | -4.628 | 0.0024 |
| front-back | mid-back | 5.019 | 0.0015 | 4.892 | 0.0018 | 6.333 | 0.0004 |
| front-back | back-back | 8.028 | 0.0001 | 18.388 | < 0.0001 | 8.156 | 0.0001 |
| mid-back | back-back | 3.359 | 0.0121 | 1.956 | 0.0914 | 2.442 | 0.0446 |

*Note.* Consonant 1 and Consonant 2 columns refer to auditory and visual place. For example, front-back refers to auditory-front/visual-back place of articulation. df = 7 for all comparison
