# Supplementary material for: Perception of incongruent audiovisual English consonants
Source: PLoS One. 2019 Mar 21;14(3):e0213588. doi: 10.1371/journal.pone.0213588 (PMC6428273; doi:10.1371/journal.pone.0213588)
Supplement: S2 Table — (DOCX) [file pone.0213588.s014.docx]

**S2 Table. Results of paired-samples t-tests comparing the proportion of visual responses across different auditory and visual places of articulation.**

| Consonant 1 | Consonant 2 | /ɑ/ t-stat, p-value | | /i/ t-stat, p-value | | /u/ t-stat, p-value | |
| --- | --- | --- | --- | --- | --- | --- | --- |
| front-front | front-mid | 3.174 | 0.0156 | -8.763 | 0.0001 | 2.047 | 0.0799 |
| front-front | front-back | 5.538 | 0.0009 | 1.839 | 0.1085 | 4.716 | 0.0022 |
| front-mid | front-back | 3.237 | 0.0143 | 14.867 | < 0.0001 | 3.160 | 0.0159 |
| mid-front | mid-mid | 1.435 | 0.1945 | 9.733 | < 0.0001 | 2.011 | 0.0842 |
| mid-front | mid-back | 3.122 | 0.0168 | 10.798 | < 0.0001 | 5.237 | 0.0012 |
| mid-mid | mid-back | 3.191 | 0.0153 | 3.290 | 0.0133 | 3.774 | 0.0069 |
| back-front | back-mid | 1.609 | 0.1517 | 3.408 | 0.0113 | 1.528 | 0.1705 |
| back-front | back-back | -0.432 | 0.6789 | 1.215 | 0.2637 | -0.552 | 0.5983 |
| back-mid | back-back | -2.263 | 0.0581 | -1.667 | 0.1395 | -0.957 | 0.3703 |
| front-front | mid-front | 1.707 | 0.1316 | -7.282 | 0.0002 | 0.108 | 0.9173 |
| front-front | back-front | 5.418 | 0.0010 | 0.874 | 0.4110 | 5.700 | 0.0007 |
| mid-front | back-front | 2.714 | 0.0300 | 9.924 | < 0.0001 | 5.302 | 0.0011 |
| front-mid | mid-mid | 0.648 | 0.5379 | 6.652 | 0.0003 | 1.070 | 0.3200 |
| front-mid | back-mid | 3.198 | 0.0151 | 17.043 | < 0.0001 | 3.893 | 0.0060 |
| mid-mid | back-mid | 4.038 | 0.0049 | 3.446 | 0.0108 | 3.665 | 0.0080 |
| front-back | mid-back | -2.693 | 0.0310 | 1.210 | 0.2654 | 2.220 | 0.0618 |
| front-back | back-back | -2.249 | 0.0593 | -1.030 | 0.3374 | 0.198 | 0.8490 |
| mid-back | back-back | -1.000 | 0.3506 | -1.468 | 0.1855 | -1.000 | 0.3506 |

*Note.* Consonant 1 and Consonant 2 columns refer to auditory and visual place. For example, front-back refers to auditory-front/visual-back place of articulation. df = 7 for all comparisons
