# Supplementary material for: Perception of incongruent audiovisual English consonants
Source: PLoS One. 2019 Mar 21;14(3):e0213588. doi: 10.1371/journal.pone.0213588 (PMC6428273; doi:10.1371/journal.pone.0213588)
Supplement: S3 Table — (DOCX) [file pone.0213588.s015.docx]

**S3 Table. Results of paired-samples t-tests comparing the proportion of Other responses across different auditory and visual places of articulation.**

| Consonant 1 | Consonant 2 | /i/ t-stat, p-value | |
| --- | --- | --- | --- |
| front-front | front-mid | -6.980 | 0.0002 |
| front-front | front-back | -3.267 | 0.0137 |
| front-mid | front-back | 4.739 | 0.0021 |
| mid-front | mid-mid | 1.301 | 0.2343 |
| mid-front | mid-back | 2.023 | 0.0828 |
| mid-mid | mid-back | 1.549 | 0.1654 |
| back-front | back-mid | 0.787 | 0.4572 |
| back-front | back-back | 0.948 | 0.3746 |
| back-mid | back-back | 3.384 | 0.0117 |
| front-front | mid-front | -2.886 | 0.0234 |
| front-front | back-front | -0.255 | 0.8059 |
| mid-front | back-front | 3.832 | 0.0064 |
| front-mid | mid-mid | 4.216 | 0.0040 |
| front-mid | back-mid | 4.579 | 0.0025 |
| mid-mid | back-mid | 4.601 | 0.0025 |
| front-back | mid-back | 2.575 | 0.0367 |
| front-back | back-back | 2.549 | 0.0382 |
| mid-back | back-back | 1.743 | 0.1249 |

*Note.* Consonant 1 and Consonant 2 columns refer to auditory and visual place. For example, front-back refers to auditory-front/visual-back place of articulation. Results are included only for the /i/ vowel, because no significant interaction of auditory and visual place was observed in the other vowel contexts. df = 7 for all comparisons
